# Do Brassica Vegetables Affect Thyroid Function?—A Comprehensive Systematic Review

**DOI:** 10.3390/ijms25073988

**Published:** 2024-04-03

**Authors:** Agnieszka Galanty, Marta Grudzińska, Wojciech Paździora, Piotr Służały, Paweł Paśko

**Affiliations:** 1 Department of Pharmacognosy, Jagiellonian University Medical College, Medyczna 9, 30-688 Kraków, Poland; agnieszka.galanty@uj.edu.pl (A.G.); wojciech.pazdziora@doctoral.uj.edu.pl (W.P.); piotr_s-95@o2.pl (P.S.); 2 Department of Food Chemistry and Nutrition, Jagiellonian University Medical College, Medyczna 9, 30-688 Kraków, Poland; marta.grudzinska@doctoral.uj.edu.pl; 3 Doctoral School of Medical and Health Sciences, Jagiellonian University Medical College, 16 Łazarza Str., 31-530 Cracow, Poland

**Keywords:** brassica, sulforaphane, indole-3-carbinol, thyroid, iodine deficiency, hypothyroidism, thyroid cancer, Hashimoto disease, goiter, vegetables, diet

## Abstract

Brassica vegetables are widely consumed all over the world, especially in North America, Asia, and Europe. They are a rich source of sulfur compounds, such as glucosinolates (GLSs) and isothiocyanates (ITCs), which provide health benefits but are also suspected of having a goitrogenic effect. Adhering to PRISMA guidelines, we conducted a systematic review to assess the impact of dietary interventions on thyroid function, in terms of the potential risk for people with thyroid dysfunctions. We analyzed the results of 123 articles of in vitro, animal, and human studies, describing the impact of brassica plants and extracts on thyroid mass and histology, blood levels of TSH, T3, T4, iodine uptake, and the effect on thyroid cancer cells. We also presented the mechanisms of the goitrogenic potential of GLSs and ITCs, the limitations of the studies included, as well as further research directions. The vast majority of the results cast doubt on previous assumptions claiming that brassica plants have antithyroid effects in humans. Instead, they indicate that including brassica vegetables in the daily diet, particularly when accompanied by adequate iodine intake, poses no adverse effects on thyroid function.

## 1. Introduction

In the human diet, brassica plants hold significant importance, offering a variety of vegetables such as various kinds of cabbage, Brussels sprouts, broccoli, and cauliflower, root vegetables like radish, turnip, and rutabaga, leafy greens including rocket or kale, as well as seasonings and relishes such as mustard and wasabi. Additionally, they serve as sources of oil, such as rapeseed oil [1]. Brassica vegetables are widely consumed across Europe, although to a lesser extent than in North America and among Asian populations. These vegetables are particularly popular in Central and Eastern Europe, as well as on the Iberian Peninsula. Historically, brassica vegetables have been suspected of negatively impacting thyroid gland function, particularly evident in livestock consuming raw or silage brassica plants, as well as in the populations where these vegetables are a significant dietary component due to their affordability and availability [2]. This negative effect was most pronounced in the regions with concurrent iodine deficiency in water and soil.

**Figure 1:**
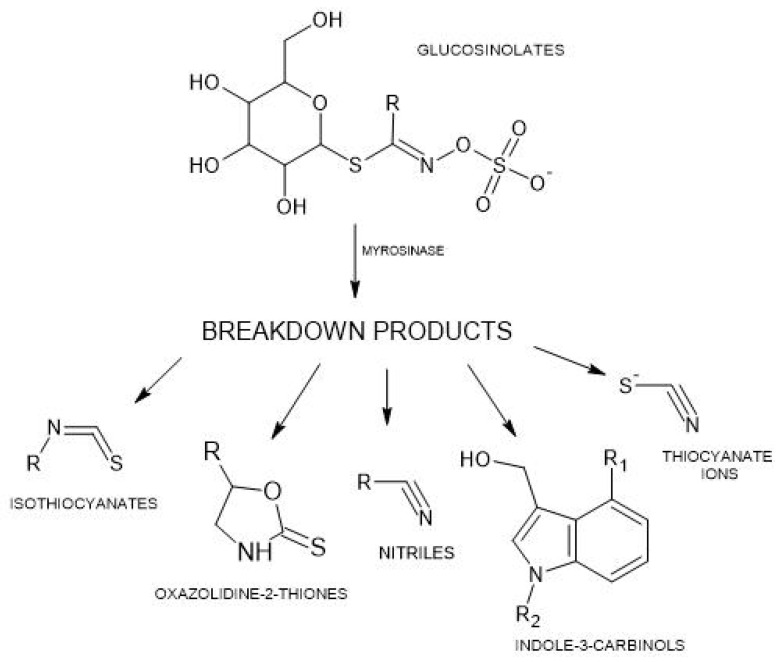
Structures of glucosinolates and their breakdown products.

The key active secondary metabolites in brassica vegetables, most often associated with antithyroid/goitrogenic effects, are sulfur compounds: glucosinolates (GLSs) and their derivatives, such as isothiocyanates (ITCs) and thiocyanates. GLSs present in brassica plants can be categorized into two groups: aliphatic, including well-known examples such as sinigrin, glucoiberin, glucoraphanin, gluconapin, and progoitrin; and indole, represented by glucobrassicin [1]. Typically, a single plant species contains up to four different GLSs in substantial quantities. The average amount of total GLSs in root vegetables is 28–330 mg/100 g fresh weight (FW), while in leafy vegetables, it ranges from 10–110 mg/100 g FW [1]. Upon ingestion, some GLSs are absorbed intact through the gastrointestinal lining, while the majority undergo metabolism within the gut. When raw brassica vegetables are chopped or eaten, the myrosinase enzyme is released from the damaged plant tissues, resulting in the breakdown of the GLSs into various metabolites, including ITCs, nitriles, oxazolidine-2-thiones, and indole-3-carbinols (Figure 1). Conversely, cooking vegetables deactivates myrosinase, allowing intact GLSs to reach the colon, where they are broken down by intestinal bacteria [3].

It is important to note that brassica vegetables also offer significant health benefits. Numerous studies have reported a significant association between higher intake of brassica vegetables and a reduced risk of cancer development, particularly in relation to cancers of the breast, prostate, lung, and digestive system. This finding highlights the potential protective effects of brassica vegetables against different type of cancers and underscores the importance of further research in thyroid cancer, which is described in the Section 3.4.

These effects are most often associated with the presence of sulforaphane (SFN) and indole-3-carbinol (I3C) in brassica vegetables, whose chemopreventive potential has been widely studied [4,5]. However, the direct relationship between individual foods and thyroid cancer is complex, and certain dietary patterns, such as consumption of fish, iodine-rich foods, vegetables, and fruits, have also been linked to a reduced risk of thyroid cancer.

The motivation behind this systematic review stems from the ongoing recommendations in dietary sources that patients, particularly those with hypothyroidism, should avoid brassica vegetables, due to their goitrogenic potential. However, these recommendations often rely on outdated animal studies, involving the seeds of these plants, which are rarely consumed by humans [6,7,8,9]. It is also important to consider that the amount of sulfur compounds in edible brassica plants vary significantly, depending on the species or even the plant part, which translates into their various impact on thyroid function.

Thus, the aim of this review is to present the published data so far, describing the goitrogenic effect of brassica vegetables, to evaluate the potential risk of their consumption and to suggest new recommendations concerning their presence in human diet. Above all, the results from human studies were taken into account, as obviously most relevant, but the data from animal studies were also included to broaden the discussed issue. Moreover, special emphasis was put on thyroid health status of the animals, or of the participants of the studies reviewed, and the form (raw or processed) of the vegetables used, as the two important factors affecting the final outcomes of the studies.

## 2. Materials and Methods

This systematic review was conducted in adherence to the PRISMA (Preferred Reporting Items for Systematic Reviews and Meta-analyses) statement. Medline, Scopus, and Google Scholar databases were searched, and the data were collected until January 2024. The keywords used were “Brassica” (and the common English names of vegetables, such as broccoli, radish), “sulforaphane”, “I3C” in combination with “thyroid”, “iodine deficiency”, “hypothyroidism”, “thyroid cancer”, “Hashimoto disease”, “goiter”. There were no time and language limits. Due to the enormous number of records in the Google Scholar database (e.g., after entering the keywords “brassica thyroid”, the search engine found 11,900 records), the method of searching by keywords contained in the title was used.

**Figure 2:**
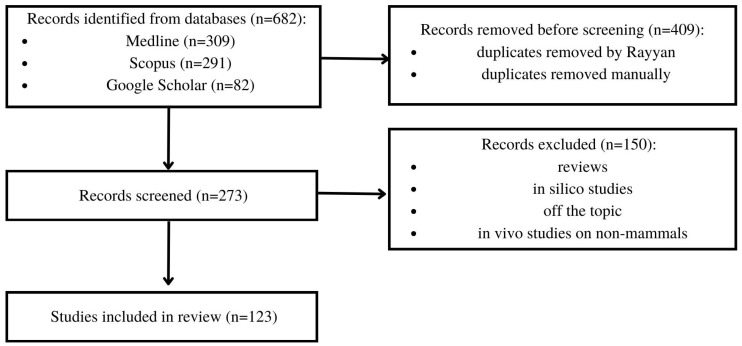
Flowchart of the search strategy.

The inclusion criteria were original articles describing the influence of brassica vegetables and isolated compounds, such as SFN and I3C on thyroid status, including serum TSH, T3, T4 levels, activity of TPO, GPX, TG, iodine uptake and thyroid weight. We considered in vitro and in vivo studies conducted on mammals, including humans. The exclusion criteria were review articles, studies on animals other than mammals, and in silico studies. The details of the search method are presented in Figure 2. In the first stage of the search, 682 articles were selected. Then, the article selection process was conducted using the Rayyan software (https://www.rayyan.ai/; accessed on 25–28 December 2023 and 30–31 January 2024), resulting in the removal of 409 duplicates. Following this, 273 articles were used for further screening, and, finally, 123 articles were selected for this systematic review.

## 3. Results

### 3.1. Impact of Brassica Sulfur Compounds on Thyroid Function

**Figure 3:**
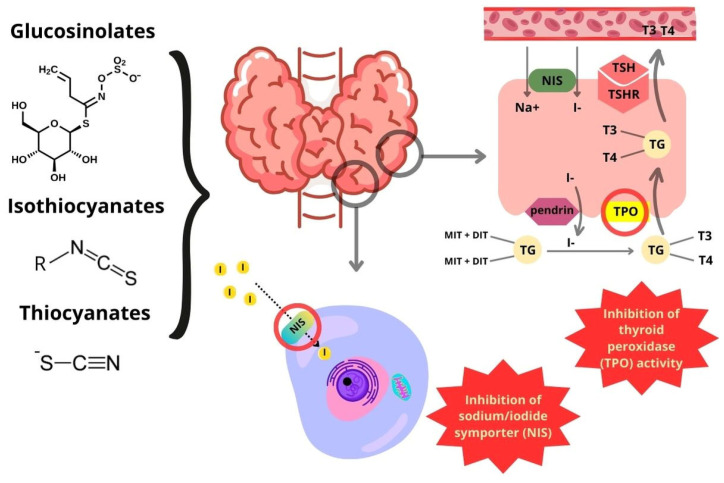
The mechanism of negative effects of glucosinolates and their derivates on thyroid cells function.

It is known that GLSs and their derivatives are potentially responsible for negative effects on thyroid function via the inhibition of the sodium/iodide symporter (NIS) in the basolateral membrane of the thyroid cells and thyroid peroxidase (TPO) activity (Figure 3).

This could predominantly result in changes in: (i) thyroid gland weight and histological appearance; (ii) iodine uptake (IU) by the thyroid glands; (iii) levels of TSH and thyroid hormones (T3, T4). These mechanisms were proved in the in vitro experiments, but also in animal and human studies, as described below. The details of the experiments are presented in Table S1.

The mechanism of the negative effect of ITCs on the thyroid function was proposed by Muztar et al., who highlighted the possible interaction of thyroxine with allyl isothiocyanate (AITC). The compound might contribute to the formation of thiohydantoin derivatives in vitro by a chemical reaction with thyroid hormones, according to the following scheme: ITCs and an amino acid may react to form a thiocarbamyl derivative, the subsequent cyclization of which may yield thiohydantoin. Another mechanism suggested is the formation of a complex of AITC with thyroxine, as a phenyl ring with an OH group at the position para to the alanine side chain is suggested to be a prerequisite for the chemical binding of AITC. These structures may decrease the ability of the organism to utilize the circulating thyroid hormones in the serum [10].

Tonacchera et al. utilized an in vitro system with human NIS, stably transfected into CHO cells to examine the simultaneous effects of perchlorate (ClO_4_^−^), thiocyanate (SCN^−^), nitrate (NO^3−^), and nonradioactive iodide (I^−^) on radioactive iodine uptake (RAIU). The primary objectives were to understand the concentration–response relationships and assess potential nonadditive interactions among these anions. Notably, thiocyanate (SCN^−^) drew special attention due to its prolonged serum half-life compared to other anions (in the serum, it is 18–29 times longer than that of the other anions). The relative potency of ClO_4_^−^ to inhibit iodine uptake by NIS was found to be fifteen times that of SCN [11].

Habza-Kowalska et al. examined the effects of ethanol extract from mustard on the activity of TPO, isolated from porcine thyroid glands. The extract acted as a competitive TPO inhibitor (IC_50_ 106.13 mg DW/mL), while after the in vitro digestion, a 24% TPO activation was observed. The authors suggested that mustard extract may contain potentially bioaccessible TPO activators, indicating potential health-promoting effects. This finding could be useful for further studies on Hashimoto’s disease [12].

A number of studies described the effect of substances with goitrogenic potential on thyroid mass, while only one study concerned the histopathological changes in the thyroid. The reported effect on thyroid mass varied, depending on the substance and animal models used.

Progoitrin, sinigrin, glucobarbarin, and sinalbin caused an increase in rat thyroid weight, with the most significant effect observed for glucobarbarin [13]. The dose-dependent increase in thyroid gland weight was also observed in a group of diabetic rats receiving AITC [14]. Several experiments showed that administering iodine together with goitrogenic substances, such as 1-methyl-2-mercaptoimidazole and potassium thiocyanate, reduced the weight of the thyroid gland in the supplemented groups of pigs, regardless of the iodine dose [15]. The opposite effect was observed for epiprogoitrin, glucoiberin, gluconapin, and glucoraphanin, as the weight of the rat thyroid glands was reduced [16].

Several studies have demonstrated that the aforementioned substances did not have any significant impact on the weight of thyroid glands in rats when administered without iodine supplementation, as observed for glucocheirolin and glucotropaeolin [16], ethylenebisisothiocyanate sulfide [17], and phenyl isothiocyanate [18].

The sole study described the influence of pure goitrogens on thyroid histopathology. In a chronic test of NN’-diethylthiourea (DETU), methylothiouracyl (MeTU), and cheiroloin on IU in rats, significant histopathological changes were noted. The first two substances caused loss of colloid and marked hyperplasia. In the case of cheiroloin, raised epithelium and colloid reduction were observed [19].

The impact of GLSs and their derivatives on the levels of TSH and thyroid hormones (T3, T4) was investigated in a number of studies. Phenyl isothiocyanate at high doses (40 mg/kg) significantly reduced the levels of T4 and FT4 in rats [18], benzyl isothiocyanate noticeably reduced the levels of rat thyroid hormones [20], while AITC decreased the levels of fT3 and fT4 in diabetic rats [14]. The effects of allyl cyanide (ACN), AITC, and dimethyl disulfide (DMDS) on thyroid hormones in lambs were described by Duncan et al., who noted a tendency to increase T3 levels for all tested compounds and a significant increase in T4 only for ACN [21]. In the experiment by Lopez-Rogriquez et al., the effect of sinigrin, along with a low iodine diet, did not have a negative impact on TSH and T3 levels in non-pregnant mares, but T4 responsiveness levels decreased after 12 weeks [22].

The antithyroid effect of thiocyanate in humans was investigated in healthy individuals with normal iodine status, who were administered a daily intake of 8 mg of thiocyanate in milk for 12 weeks. The study revealed significant increases in serum thiocyanate levels, peaking at 4 weeks, with a rise of +3.8 mg/L. Simultaneously, urine excretion also saw a notable increase of +4.8 mg/g creatinine. Despite these changes, the initial levels of T4, T3, and TSH remained within normal ranges throughout the study period [23].

Bruce et al. assessed the potential impact of urinary concentrations of iodine uptake inhibition (IUI) agents, including nitrate, perchlorate, and specifically thiocyanate, on serum thyroid parameters. The research utilized enhanced thyroid hormone measures from the National Health and Examination Survey (NHANES) 2001–2002 dataset. Multiple regression analysis was employed to evaluate the relationships between total thyroxine (TT4), free thyroxine, total triiodothyronine (TT3), free triiodothyronine, TSH, and perchlorate equivalent concentration (PEC), with a focus on thiocyanate. The results revealed a weak and negative association between PEC and TT4, primarily influenced by nitrate and thiocyanate. However, no significant associations were observed with TSH, TT3, or free hormones. Despite exposure to the combined IUI agents, the analysis did not provide evidence of functional thyroid abnormalities, such as low thyroid hormone coupled with high TSH [24].

The possible negative influence of different doses of progoitrin on the thyroid was evaluated in humans using acute inhibition of radioiodine uptake, as well as 24 h uptake, by Greer et al. The same authors also assessed the hydrolysis of progoitrin into goitrin in the large intestine through rectal administration of this compound. It should be noted that progoitrin was also tested as an antithyroid agent in one patient with Graves’ disease, in comparison to propylthiouracil [25].

A study by Rajoria et al. confirmed that sulfur compounds can be distributed and detected in the human thyroid and may present a strong impact on this gland. In a phase I clinical trial of patients diagnosed with thyroid proliferative disorders, 3,3′-diindolylmethane (DIM) was administered in a daily dose of 300 mg for 14 days prior to undergoing thyroidectomy. Tissue, urine, and serum samples were collected both before and after DIM supplementation. The analysis revealed the presence of DIM in thyroid tissue, serum, and urine, affirming its successful delivery to target areas [26].

### 3.2. Studies Based on Surveys/Questionnaires

Apart from the mechanistic data on the impact of the brassica sulfur compounds on the thyroid, a number of survey studies suggest some relationship between the consumption of brassica vegetables and thyroid dysfunction (Table S2). One of the first studies published concerned iodized rapeseed oil, whose consumption by patients with different stages of goitrous swelling decreased the thyroid size to normal after 90 days. Moreover, on the 30th and 90th day of the study, all thyroid function indicators stabilized and returned to normal values [27].

The dietary patterns amongst school children of the Howrah district in Gangetic West Bengal were investigated to examine the effect of dietary goitrogens and their impact on iodine deficiency disorders, which are reported in this area despite the introduction of iodized salt. Based on the thiocyanate concentration in urine samples, it was found that the children consumed a number of brassica vegetables. No iodine deficiencies were found in the population studied. Moreover, it was shown that the ratio of iodine to thiocyanates in urine in the study area was much above the critical level, which may suggest that a diet rich in brassica vegetables does not necessarily contribute to thyroid disorders [28]. In a similar study with primary school girls in Qom city of Iran, authors conclude that the main risk factor for goiter was improper salt storage in open containers, while the history of kale and turnip consumption for the control and goiter groups did not show any significant differences [29].

Alissa et al. [30] studied the effect of a diet rich in goitrogens on indices of iodine and thyroid status in people living in Jeddah, Saudi Arabia. The results showed that the difference in thyroid parameters between hypothyroid patients and healthy people was not statistically significant. Food rich in goitrogens, especially turnip included in the diet, was associated with lower urinary iodine concentration (UIC). Another observation was a positive association between UIC and serum TSH level, and a negative association with serum T4 level. However, the concomitant levothyroxine therapy in the vast majority (85%) of the patients might have influenced the results. Imdad et al. [31] evaluated the impact of dietary patterns on the thyroid status of the adolescent girls’ population in City District, Lahore. Almost 9% of all participants had goiter, related to the consumption of cabbage, but not cauliflower or turnip. Similarly, cabbage consumption at least once a week was associated with goiter in primary school children in the Anchar district, Eastern Ethiopia, in which the total goiter prevalence rate was very high [32].

The survey conducted by Hason et al. found that 88.4% of the 500 participants diagnosed with hypothyroidism consumed cabbage, which might promote the development of thyroid diseases. However, lack of a control group of healthy subjects and methodology details make the results questionable [33]. A similar study with participants with suspected thyroid disorders living in Algeria, showed no connection between the consumption of brassica vegetables rich in GLSs and the occurrence of goiter, but indicated that the condition of people suffering from this disease may worsen. Among the examined participants, the incidence of goiter was found to be 17.5%, and the consumption of brassica vegetables was 54.32 g per person per day, which translates to 52.7 µmol GLSs per day [34]. A recent questionnaire study from Poland on people on traditional, vegetarian, vegan, and pesco-vegetarian diets (n = 365) conducted a correspondence analysis model which did not show a relation between brassica vegetables consumption and thyroid dysfunction [35].

### 3.3. Influence of Brassica Vegetables on Animal and Human Thyroid

The details of the conducted animal and human studies for particular brassica vegetables are presented in appropriate tables, while a brief summary of the results is described below.

#### 3.3.1. Broccoli

Although broccoli is one of the most popular brassica vegetables in the daily diet, surprisingly few data exist on its impact on thyroid function (Table 1). Experiments on ewes and lambs with non-dysfunctional thyroid, fed with broccoli stems and leaves, indicated no significant changes in thyroid function when compared to the control group [36,37]. On the other hand, broccoli sprouts administered to rats with sulfadimethoxine-induced thyroid dysfunction counteracted the toxic effect of the compound by decreasing TSH and FT3. Additionally, an increase in the enzymatic activity of the thyroid gland was observed, as the normalized levels of glutathione peroxidase and thioredoxin reductase were noted [38]. In the follow-up study by the authors, the use of the broccoli sprouts diet resulted in a tendency to reduce the frequency of negative histopathological changes in the group with thyroid dysfunction [39].

A case report described a patient with symptoms such as muscle aches and pains, initially diagnosed as rhabdomyolysis, but after further evaluation as Hashimoto disease. In the interview, the patient mentioned consuming large servings (although not specified) of raw broccoli and alcohol in the past 2 weeks. He was treated with levothyroxine and was told to avoid alcohol and goitrogenic foods such as broccoli. After a 2-month follow-up the patient was asymptomatic, and his blood reports showed normal thyroid parameters. This may suggest that overconsumption of raw broccoli can cause thyroid dysfunction. However, due to the treatment with levothyroxine it cannot be clearly concluded that the thyroid improvement resulted from avoiding broccoli consumption [40].

A few dietary intervention-based studies in healthy human subjects concerned broccoli sprouts consumption. In a double-blind, placebo-controlled, randomized clinical study, broccoli sprouts extracts containing either GLSs or ITCs, administered to healthy subjects caused notable but not statistically significant changes in TSH levels, which suggests their safety in relation to thyroid status [41]. Similar observation was made by Yanaka et al., [42], where daily intake of 20 g of broccoli sprouts for 4 weeks by healthy human subjects did not significantly influence TSH, fT3, and fT4 levels when compared to the control group. Moreover, no hypothyroid symptoms were observed in the participants during the study period. A long-term (12 weeks) exposure to a broccoli sprouts beverage enriched with glucoraphanin and SFN on thyroid function in healthy subjects did not affect the serum levels of TSH, T4, or thyroglobulin (TG). The thyroid autoimmunity status of the participants was also not affected, as compared to the placebo group [43].

**Table 1:** Goitrogenic potential of broccoli—animal and human studies.

Plant Material	Animals/ Patients	Study Design	Results	References
		Animal Studies		
broccoli sprouts (*Brassica oleracea* L. convar. *botrytis* var. *cymosa*) (113.33 ± 12.58 mg/100 g DW. SFN)	male Wistar rats (n = 72)	Groups: (1) control; (2) broccoli sprouts diet; (3) iodine-deficient diet; (4) iodine-deficient diet with broccoli sprouts; (5) standard diet with 0.025% SDM; (6) broccoli sprouts diet with 0.025% SDM Procedures: tests for the level of thyroid, cholesterol, morphology, liver, and antioxidant levels Time: 8 weeks	TSH, fT3—protective effect of sprouts against SDM no change in thyroid hormone levels between control and broccoli sprouts group ↑ thioredoxin reductase (mU/mg) in group 2 and group 4 vs. control (7.13 ± 1.28, 5.73 ± 1.65, 2.53 ± 0.13, respectively) in thyroid glands	[38]
broccoli by-product (BB)-wheat straw (WS) silage (BBWS; 69:31 ratio DM)	male Fashandy lambs; average BW 29.9 ± 0.9 kg; 6 months old (n = 45)	Groups: (1) diet with 0% BBWS; (2) diet with 10% BBWS; (3) diet with 20% BBWS Procedures: blood sample was taken to check thyroid hormones. Time: 10 weeks	thyroid hormones were not affected by intervention of broccoli	[37]
broccoli (*Brassica oleracea* L. var. *italica* cv. *Naxus*) (inflorescences, leaves, stems powder)	FVB/N male mice (n = 10)	Groups: (1) control; (2) 6.7% broccoli by-product flour Procedures: analysis of internal organs weight, liver and kidney oxidation parameters and selected biochemical indicators Time: 21 days	no differences in body weight, cholesterol, ALT, AST levels and CAT, SOD, ROS, GSH in liver and kidneys	[44]
broccoli sprouts (*Brassica oleracea* L. convar. *botrytis* var. *cymosa*) (113.33 ± 12.58 mg/100 g DW. SFN)	Wistar male rats (n = 72)	Groups: (1) control; (2) broccoli sprouts diet; (3) iodine-deficient diet; (4) iodine-deficient diet with broccoli sprouts; (5) standard diet with 0.025% SDM; (6) broccoli sprouts diet with 0.025% SDM Procedures: histopathology analysis Time: 8 weeks	tendency to ↓ changes in overall thyroid function with the addition of sprouts in groups with thyroid dysfunction (3.50 ± 1.13 in 3 vs. 2.67 ± 0.6 in 4, 4.64 ± 1.40 in 5 vs. 4.42 ± 0.63 in 6) and follicular epithelial (0.69 ± 0.24 in 3 vs. 2.67 ± 0.6 in 4, 4.64 ± 1.40 in 5 vs. 4.42 ± 0.63 in 6) areas (mm^2^)	[39]
broccoli waste (leaves and stems)	Kurdi ewes, aged 2–3 years (n = 12)	Groups: (1) control diet without broccoli; (2) diet with 250 g of broccoli; (3) diet with 750 g of broccoli Procedures: thyroid function checked by blood sample with biochemical parameters and thyroid hormones Time: 8 weeks	no significant changes in thyroid hormones and weight of animals	[36]
		Human Studies		
broccoli	technicians, medical students, and physicians (no data about health status) (n = 100)	Procedures: subjects with no breakfast, given a tracer dose of 100 microcuries of radioactive iodine in physiological saline; the uptake of radioactive iodine by the thyroid determined, followed by the instruction for the subjects to eat certain foods, such as cruciferous vegetables	lack of effect of cooked broccoli on the iodine uptake	[7]
broccoli sprouts	healthy volunteers (n = 12) randomized, placebo-controlled, double-blind trial	Groups: 3 cohorts of 4 people, 2 groups each: (1) an extract from broccoli sprouts (n = 3); (2) a matching placebo extract (n = 1). Procedures: After a 5-day acclimatization period, the subjects received orally for 7 days broccoli sprout extracts containing either GLSs or ITCs in the doses: cohort A: 25 μmol of GLSs; cohort B: 100 μmol of GLSs; cohort C: 25 μmol of ITCs	TSH levels exceeded the upper limits of normal during or after the dosing period (4.4–7.6 μIU/mL)	[41]
broccoli sprouts	healthy volunteers (n = 48) placebo-controlled semi-open label intervention trial	Groups: (1) 20 g of raw alfalfa sprouts without SFN GLSs (n = 24); (2) 20 g of raw broccoli sprouts (4.4 mg/g SFN GLSs, (n = 24) Procedures: 2-week pre-trial period, the 4-week intervention period, and the 4-week post-trial observation period. Blood samples collected and screened for TSH, T3, T4 values	alfalfa sprouts—TSH 2.05–2.34 mIU/mL, fT3 3.04–3.07 pg/mL, fT4 1.24–1.26 ng/dL broccoli sprouts-TSH 1.92–2.14 mIU/mL, fT3 2.98–3.25 pg/mL, fT4 1.2–1.28 ng/dL	[42]
broccoli sprouts	healthy subjects (n = 267) randomized trial	Groups: (1) broccoli sprout beverage (n = 137); (2) placebo beverage (n = 130) Procedures: Broccoli sprouts beverage containing 40 μmol SFN and 600 μmol of glucoraphanin administered for 12 weeks. Blood samples from day 0 and day 84 (last day) of the study collected. Serum TSH, free thyroxine (fT4), thyroglobulin (TG), anti-TG, and antithyroid peroxidase (anti-TPO) antibodies measured in samples from 45 female participants	placebo—TSH 4.58 ± 0.47 mIU/L, fT4 1.51 ± 0.05 ng/dL, anti-TG and/or anti-TPO seropositivity 58% broccoli sprouts—TSH 3.8 ± 0.6 mIU/L, fT4 1.59 ± 0.04 ng/dL, anti-TG and/or anti-TPO seropositivity 42%	[43]
Broccoli	patient with Hashimoto disease (n = 1) case report	Procedures: patient consuming large servings of raw broccoli was diagnosed with Hashimoto’s disease and prescribed levothyroxine 50 μg/day; he was advised to avoid alcohol and goitrogenic foods like broccoli. Patient was screened for TSH, T3, T4 values before and after diagnosis	before—TSH 68 μIU/mL, fT3 20.6 ng/dL, fT4 0.88 μg/dL, and positive antithyroid peroxidase antibody of 2372 U/mL after—normal thyroid parameter, no data available	[40]

↑↓: Statistically significant increase or decrease in values.

#### 3.3.2. Brussels Sprouts

Only two studies reported the effect of Brussels sprouts on the thyroid (Table 2). In rats with non-dysfunctional thyroids, fed with raw or cooked Brussels sprouts, a decrease in the level of thyroxine in the serum was observed, but there was no change in TSH concentration, regardless of the cooking process [45]. McMillan et al. evaluated the effect of boiled or steamed Brussels sprouts, rich in GLSs (220 mg/100 g), on thyroid function in healthy human subjects. The results showed no significant changes in serum TSH, T4, or T3 levels, irrespective of the presence of Brussels sprouts in the diet. The authors suggested that goitrin, present in Brussels sprouts, was deactivated due to the cooking process, and therefore, a diet rich in Brussels sprouts did not affect the thyroid [46].

#### 3.3.3. Cabbage

Despite the high popularity of cabbage in the human diet, only two studies reported its effect on humans, while the majority of studies reported the impact of different varieties of the vegetable on animal thyroid function (Table 3). Miller et al. [56] found that a 20% share of cabbage in the diet had no effect on thyroid weight in rats. Similarly, Goldie et al. used different proportions of cabbage and millet in rat feeds. In the experimental groups, where the share of cabbage juice was 30%, the greatest increase in TSH over 90 days was observed when 30% of millet was consumed in the diet. When 60% cabbage juice was used in all dietary treatments, the hormone level increased, but no significant differences were noted [57].

Rabbits fed with fresh juice of winter cabbage for 3 weeks developed a simple goiter, with the size of the thyroid being twice the normal size, and marked hyperemia was observed in the intervention group, while in the group fed with red cabbage, a 1.5-time increase in thyroid gland size and moderate hyperemia were noted [48]. Likewise, a significant increase in the thyroid gland was noted in lambs fed with Chinese cabbage, compared to the animals supplemented with additional iodine. This suggests that iodine supplementation might neutralize the goitrogenic effect of the plant [55]. On the other hand, a study on lambs fed with chopped grass hay and tyfon (a turnip x Chinese cabbage hybrid) did not show any statistical changes in thyroxine and triiodothyronine levels in plasma; only daily weight gain was seen in intervention groups [58].

An interesting study compared the goitrogenic impact of dry, powdered cabbage and pure thiocyanate, added to the diet of rats, which were additionally supplemented with iodine in the form of potassium iodide. An increase in thyroid weight in rats on a diet containing 33% cabbage was noted, and the effect was enhanced in the group additionally supplemented with iodine. Moreover, the cabbage diet caused a significant decrease in serum T4 concentration compared to the control group, regardless of iodine supplementation. Interestingly, the level of T4 increased, and the weight of the thyroid gland decreased after iodine supplementation in rats taking thiocyanates compared to the cabbage-fed group. Moreover, the use of iodine together with thiocyanate enabled the maturation of follicles in the thyroid gland, which suggests hypothyroidism. In the cabbage group, no differences were observed between the presence or absence of iodine in the semi-quantitative assessment of thyroid follicles [59].

Two studies described the effect of cabbage in rat poisoning models. In cadmium (II) chloride poisoning, the use of cabbage extract intensified the decrease in T3 and T4 levels compared to the poisoned control group. It is worth mentioning that the extract stimulated the formation of GSH, SOD, CAT, which are responsible for detoxification processes. However, feeding rats only with cabbage extract caused a decrease in thyroid hormone values compared to the control group [60]. Hamouda et al. [61], in turn, observed that administering cabbage extract in the case of tetrachloromethane poisoning deepens its toxic effect in rats. A greater decrease in thyroxine and triiodothyronine was found, compared to the poisoned group.

As far as human dietary intervention-based studies are concerned, one of the oldest studies found concerned the evaluation of the antithyroid effect of the selected brassica vegetables, determined by the uptake of radioactive iodine by the thyroid gland after consumption. The consumption of cabbage did not cause significant antithyroid activity [7].

A case study of a Chinese woman with diabetes described t several months of overconsumption of raw Chinese cabbage, up to 1.5 kg a day, which was associated with the traditional Chinese medicine indication for the plant as reducing blood glucose levels. Laboratory tests showed an elevated level of TSH and undetectable free thyroxine. The woman was diagnosed with severe hypothyroidism [62].

**Table 3:** Goitrogenic potential of cabbage—animal and human studies.

Plant Material	Animals/ Patients	Study Design	Results	References
		Animal Studies		
winter cabbage	rabbits of both sexes, aged 5–6 months, average weight of 2.2 kg (n = 12)	Groups: (1) 300 mL of fresh juice of cabbage; (2) 200 mL of fresh juice of cabbage; (3) control group alfalfa hay (each group n = 4) Procedures: condition of thyroid and weight of thyroid gland Time: 3 weeks	size nearly twice normal size—moderate hyperemia development of simple goiter in rabbits was found	[48]
red cabbage	rabbits of both sexes, aged 5–6 months, average weight of 2.2 kg (n = 8)	Groups: (1) red cabbage 465 g daily; (2) control group alfalfa hay (each group n = 4) Procedures: condition of thyroid and weight of thyroid gland Time: 7 weeks	1.5 times normal size of the thyroid gland—moderate hyperemia	[48]
20% cabbage diet (*Brassica oleracea* var. *excel*) (freeze-dried ground cabbage)	male Fischer (F), Long-Evans (LE), Sprague- Dawley (SD) rats (n = 96)	Groups: (1) control F rats; (2) 20% cabbage F rats; (3) control LE rats; (4) 20% cabbage LE rats; (5) control SD rats; (6) 20% cabbage SD rats Procedures: analysis of the weight of internal organs and liver parameters Time: 3–4 weeks	no differences in thyroid weight between groups after 4 weeks.	[56]
thiocyanate (SCN) cabbage (CAB) (cabbage powder)	female rats of Wistar/NIN (n = 6–8 in each group)	Groups: (1) G1 (+KI (316 µg KI/10 g); (2) G2 (-KI (3 µg KI/10 g); (3) G3 (SCN (25 mg)—KI); (4) G4 (SCN + KI); (5) G5 (CAB (1/3 of the feed weight)—KI); (6) G6 (CAB + KI). Procedures: assessment of the weight of internal organs, thyroid, urine parameters, histological assessment Time: 60 days	↓ T4 level (µg/dL) in G3, G5, G6 groups (3.91 ± 0.33, 3.75 ± 0.17, 3.72 ± 0.20, respectively, vs. 6.2 ± 0.27 in G2) ↑ thyroid weight (mg/100 g b.w.) in G3, G5 groups (6.64 + 0.45; 6.45 ± 0.52, respectively, vs. 4.17 ± 032 in G2) no differences in the histological image of the thyroid gland between groups G5 and G6	[59]
cabbage extract	adult male Wistar albino rats (n = 28)	Groups: (1) control; (2) cabbage extract group (5 mL/kg b.w/day; (3) CdCl_2_ group (100 mg/L) (4) cabbage + CdCl_2_ group Procedures: analysis of thyroid hormone levels, glucose, kidneys, and liver profiles Time: 4 weeks	↓ T4 in all experimental groups—the most in combined group (77.35 ± 4.9 vs. 113.42 ± 4.84 nmol/L in control) ↓ T3 in all experimental groups—the most in combined group (3.78 ± 0.43 vs. 5.88 ± 0.26 nmol/L in control) protective effect of cabbage extract against CdCl2—↑ GSH, SOD, CAT in combined group vs. CdCl_2_ group	[60]
millet supplement and cabbage juice	albino rats (n = 60)	Groups in Experiment 1 (E1): (1) control; (2) 30% millet; (3) 60% millet; (4) 100% millet Groups in Experiment 2 (E2): (1) 30% cabbage; (2) 30% millet+ 30% cabbage; (3) 60% millet+ 30% cabbage Groups in Experiment 3 (E3): (1) 60% cabbage; (2) 30% millet+ 60% cabbage; (3) 60% millet+ 60% cabbage Procedures: analysis of thyroid hormone levels Time: 90 days	↑ TSH in group 1 (60% cabbage) in 90 days (0.41 ± 0.32 → 2.34 ± 0.35 mIU/mL) in E3 ↑ TSH in group 1 (30% cabbage) in 90 days (0.34 ± 0.22 → 1.83 ± 0.20 mIU/mL) in E2	[57]
avocado extract (homogenized pulp) *Zingiber officinale* extract (ethanolic extract of ginger) *Brassica oleracea* extract (ethanolic extract of cabbage)	adult male Sprague-Dawley rats (n = 80)	Groups: (1) control; (2) CCl_4_ group (3 mL/kg per week for three weeks); (3) avocado group (1 mL/kg b.m. daily); (4) avocado + CCl_4_ group; (5) ginger group (30 mg/kg b.m.); (6) ginger+ CCl_4_ group; (7) cabbage group (500 mg/kg b.m.) (8) cabbage + CCl_4_ group Procedures: tests of the level of thyroid hormones, lipid and liver profiles, microscopic analysis of tissue Time: 35 days	no changes in T3, T4, TSH level in pure extracts groups vs. control ↑ toxicity CCl_4_ after cabbage—↓ T3, T4 in combined group vs. CCl_4_ group (0.30 ± 1.23 ng/mL, 8.34 ± 1.01 µg/dL vs. 0.40 ± 2.25 ng/mL, 12.96 ± 4.45 µg/dL, respectively) no protective effect of cabbage extract against CCl_4_—↑ NO in liver, ↓ MDA in combined group vs. CCl_4_ group	[61]
fresh cabbage	male Wistar rats (n = 56)	Groups: (1) control; (2) iodine deficiency; (3) iodine deficiency + undernutrition; (4) iodine deficiency + thiocyanate; (5) iodine deficiency + thiocyanate + undernutrition; (6) propylthiouracil; (7) cassava diet + distilled water; (8) brassica diet + distilled water; (9) Remington + iodized water + selenium; (10) Remington + iodized water; (11) Remington ration supplemented with selenium; (12) iodine deficiency + calcium + distilled water; (13) millet diet + distilled water; (14) Nétu diet + distilled water Procedures: measurement of body weight, water, and food intake Time: 120 days	highest food intake and lowest water intake in brassica diet group ↑ body weight (g) in brassica diet group vs. control (241.25 ± 14.08 vs. 172.50 ± 05.35), but not the largest of all groups	[63]
		Human Studies		
cabbage	technicians, medical students, and physicians (no data about health status) (n = 100)	Procedures: subjects with no breakfast, given a tracer dose of 100 microcuries of radioactive iodine in physiological saline; the uptake of radioactive iodine by the thyroid determined, followed by the instruction for the subjects to eat certain food such as cruciferous vegetables	lack of effect of raw cabbage on the iodine uptake	[7]
*Brassica rapa chinensis* (raw bok choy)	patient with hypothyroidism (n = 1) (case report)	Procedures: case report. An 88-year-old diabetic patient was diagnosed with hypothyroidism after consuming 1–1.5 kg of bok choy a day and treated intravenously with levothyroxine and methylprednisolone. The patient was examined for TSH, TPO Ab and fT4 values, body temperature, blood pressure and pulse were measured.	TSH 74.4 mIU/L (0.65 mIU/L 4 months earlier), fT4 undetectable, thyroid peroxidase antibodies (13 IU/mL), pressure 181/89 mm Hg, pulse 58 beats/min, temperature 36.1 °C	[62]

↑↓: Statistically significant increase or decrease in values.

#### 3.3.4. Cauliflower

A single study described the protective potential of cauliflower against toxins (Table 2). In a fluoride poisoning rat model, the extract at a dose of 200 mg/kg shows the greatest potential to reduce the effects of fluoride poisoning. At the same time, the administration of the extract at a dose of 400 mg/kg to the group increased the level of thyroxine in rat serum [47].

Cooked cauliflower showed no effect on the uptake of radioactive iodine by the thyroid in humans [7].

#### 3.3.5. Kale

The goitrogenic potential of kale was investigated in a number of animal studies (Table 4). A study performed on guinea pigs fed with kale and radioiodine indicated lower accumulation of the element in thyroid of the animals on the kale diet when compared to the control group. The authors thus suggest a goitrogenic effect of kale [49]. Barry et al. observed an almost four-time decrease in triiodothyronine levels in cattle fed with kale, while the supplementation with iodine resulted in a normalization of thyroid gland weight and thyroid hormones [64]. Similarly, Duncan et al. observed that a low-iodine diet in lambs fed with fresh kale may lead to thyroid dysfunction, observed as thyroid enlargement and a significant reduction in thyroid hormones (almost 90% reduction in thyroxine levels) [65]. This was also confirmed by further studies on lambs fed with kale alone or with kale supplemented with iodine. An increase in the thyroid gland was significant in the animals fed with kale compared to the iodine-supplemented group, where the thyroid weight did not change. This suggests that iodine supplementation might neutralize the goitrogenic effect of kale [55].

A high ratio of goiter appearance, over 60%, was observed in ewes fed with fresh kale when compared to the group fed with other pastures, where the percent of goiter incidence was almost three times lower [66].

**Table 4:** Goitrogenic potential of kale—animal studies.

Plant Material	Animal Model	Study Design	Results	References
kale consumed at a rate of 70–80 g/day	guinea pigs (n = 12)	Groups: (1) control group fed with carrots; (2) kohlrabi fed; (3) kale fed (each group n = 4) Procedures: iodine concentration in thyroid gland after 24 h after fed Time: 1 day	in kale group significant lower iodine 131 concentration in thyroid gland in kale group 2.778 ± 636 c.p.m/mgm, in control 5.549 ± 994 c.p.m/mgm.	[49]
kale (in the form of marrow stem)	Welsh mountain ewes four-and-a-half-years old (n = 36)	Groups: (1) hay ad libitum—control; (2) kale fertilized with nitrochalk, ad libitum; (3) kale fertilized with nitrate of soda, ad libitum (each group n = 12) Procedures: plasma total iodine concentration and thyroid weight iodine concentration and histological appearance were observed before and after the intervention Time: 6 weeks	plasma total iodine ↓ (−39.47% (2) and −47.62% (3) vs. control −4.65% (1)) thyroid iodine concentration ↓ (−60.71% (1) and −78.69% (3) vs. control −36.76% (1)) thyroid weight no significant change	[67]
kale (*Brassica oleracea* var. *acephala*)	healthy N’elsh mountain ewes (n = 40)	Groups: (1) kale ad lib.(n = 10); (2) kale ad lib +0.22 kg blood meal/flaked maize (n = 11); (3) kale ad lib. +0.22 kg blood meal/flaked maize and 10 mg of potassium iodide weekly(n = 8); (4) control group 0.9 kg hay +0.22 kg blood meal/flaked maize (n = 11) Procedures: at the end of the feeding period, sheep were killed and thyroid glands examined for histological purposes; iodine concentration and thyroid weight were measured Time: 17 weeks	only the group receiving kale showed increase in thyroid weight (+121%) (3) vs. control (0%) sheep fed on kale developed thyroid enlargement, which decreased after iodine supplementation	[68]
kale (*Brassica oleracea* var. *acephala*, 10.5 kg/head/d.) sulfur (% DW) content 0.80 ± 0.104 (control pasture: 0.45 ± 0.094) GLSs were present in the kale (11 uM/g DW.)	(Friesian x Angus) steers (n = 64)	Groups: (1) kale (n = 16); (2) kale and copper (n = 16); (3) kale + iodine and copper supplementation (n = 16); (4) feeding with pasture (n = 16) Procedures: blood samples were taken to examine biochemical and hormonal parameters Time: 24 weeks	thyroid hormones ↓ (T4 +5.16%/0% (4), T3 −24.39% (1) vs. control −3.21% (4), T4 −2.76/0% (2), T3 −32.43% vs. control 0% (2), T4 + 5.53% vs. control 0% (4), T3 −13.51% vs. control 0% (4) weight of thyroid gland ↓ −24.71% (1) vs. control 0% requirements for co-supplementation of copper with kale-based diet only for periods exceeding 12 weeks	[64]
kale (20 pg iodine/kg dry matter (DM) and 8 pmol total GLSs/g DM and 11.5 g S-methyl-L-cysteine sulfoxide (SMCO)/kg DM)	Romney wether lambs aged 5 months and with a mean initial live weight of 23.6 kg. (SD 2.2) (n = 96)	Groups: factorial experiment conducted utilizing two diets (kale and ryegrass-clover pasture), two rates of I supplementation (with and without) and two rates of copper supplementation (with and without, every group n = 12) Procedures: histopathological assessment was performed at the end of intervention Time: 24 weeks	serum level of thyroid hormones ↓ (T4−89.87% vs. control−1.24%) in non-iodine/copper group thyroid enlargement and depletion of thyroid was confirmed diet without iodine leads to dysfunction of thyroid and hormones when fed with kale supplementation of iodine was required if sheep were fed with kale	[65]
kale (*B. oleracea* L. var. *acephala* DC)	crossbred Suffolk-Dorset lambs (50 wethers, 50 ewes, weighing 30 to 40 kg) (n = 100)	Groups in Experiment 1 (E1): (1) no supplementation; (2) iodine supplementation; (3) copper supplementation; (4) iodine plus copper Groups in Experiment 2 (E2): (1) pasture as control; (2) kale-diet Procedures: postmortal extraction of thyroid gland measured for weight Time: 17 weeks	thyroid gland weight ↑ (+40.67% (1)/+ 10.2% (4) (E1) average body weight gain (+67 g (kale)/+89 g daily (E2)) confirmed enlargement was modest	[55]
grazed paddocks of brassica kale (*Brassica* *oleracea* spp. *acephala*)	mixed-aged Romney ewes (n = 350)	Groups: (1) control (n = 175); (2) kale (n = 175) Procedures: the ewes were supplemented pre-mating or at the time of pregnancy scanning with an injection of long-acting iodized oil. Serum iodine concentrations were measured. The thyroid weight-to-birthweight ratios (as g/kg) in 229 newborn lambs were determined at post-mortem examination and compared between iodine supplemented vs. unsupplemented flocks Time: 29 weeks	clearly indicative of goiter, including 62% (2) from the groups on brassica vs. 18% (1)	[66]
forage kale (*Brassica oleracea* spp. *acephala*)	lightweight calves—Angus-Simmental crossbred weaned bull calves (6 to 7 months old) (n = 40)	Groups: (1) (control) low nutritive value pasture and hay plus 1 kg/d of rolled barley; (2) (pasture) management intensive pasture; (3) (haylage) timothy haylage; (4) (kale) 50% timothy haylage −50% kale pasture. Procedures: blood samples were analysed for thyroid hormones Time: 4 weeks	thyroid hormones ↑ (T3 + 65.25% (4) vs. control − 3.07%, T4 + 18.23% (4) vs. control + 10.97%) daily weight gain (−28.36% (4) vs. control − 2.65%)	[69]

↑↓: statistically significant increase or decrease in values.

#### 3.3.6. Kohlrabi

Only three studies reported the impact of kohlrabi on thyroid function (Table 2). Kohlrabi sprouts fed to rats with sulfadimethoxine-induced thyroid disorders revealed a tendency for a protective effect against TSH and T3 hormones in groups with thyroid dysfunction and for reducing negative changes in the morphological image of the thyroid gland [50].

A study performed on guinea pigs fed with kohlrabi and radioiodine indicated lower accumulation of the element in the thyroid of the animals when compared to the control group, which may suggest the goitrogenic potential of the plant [49]. Another research was conducted on rabbits [48], fed with kohlrabi for 5 weeks. The results revealed a significant outcome in the thyroid gland, which was twice the normal size, and moderate hyperemia was confirmed.

#### 3.3.7. Mustard

A number of studies concerned mustard and its by-products, the majority of which indicated a significant reduction in thyroid hormones and an increase in the size of the thyroid gland (Table 5).

Giving the mustard seed oil to rats with thyroid dysfunction appeared to normalize its function. In rats with hyperthyroidism, there was a noticeable decrease in thyroxine and triiodothyronine, with an increase in TSH. The opposite situation was observed in rats with hypothyroidism: an increase in thyroxine and triiodothyronine, with a simultaneous decrease in TSH levels. There was also a dose-dependent effect, where the dose of 2 mL/kg turned out to be more effective than 1 mg/kg. In both oil-fed groups, a decrease in the liver enzymes ALT, AST, and ALP was also observed [70].

Al-Divan et al. showed the protective potential of mustard seed oil in cadmium poisoning [71]. In this study, the oil alleviated the toxic effects of cadmium (II) chloride, counteracting the decrease in T3 and T4, but these values were still significantly lower than in the control group. The oil has proven to be effective in preventing damage to the kidneys, liver, and hematopoietic system. It is worth mentioning that the use of mustard oil alone increased the levels of TSH, thyroxine, and triiodothyronine.

Pattanaik et al. studied the goitrogenic potential of mustard cake, included into the diet of goats, with or without iodine supplementation. The results confirmed a goitrogenic effect in animals fed with mustard cake, observed as the decreased levels of thyroid hormones, but the effect was neutralized by supplementation of iodine [72]. Similarly, Tripathi et al. evaluated the effect of a mustard meal-based diet and co-administration of iodine on the function of the thyroid gland. The supplementation of iodine normalized the level of hormones and weight of the thyroid gland compared to the intervention groups [73].

A sole study described the effect of Ethiopian mustard fed to heifers. The results revealed a significant reduction in concentrations of triiodothyronine in the intervention group compared to the control group [74].

**Table 5:** Goitrogenic potential of mustard—animal studies.

Plant Material	Animal Model	Study Design	Results	References
30% of mustard (*Brassica juncea*) cake concentration and ad libitum oat hay	2-year-old male Adult barbari goats (average weight 18.8 kg) (n = 12)	Groups: (1) control with 0 mg of iodine; (2) diet with 0.05 mg of iodine; (3) diet with 0.075 mg of iodine. All groups were fed with mustard cake. Procedures: measurement of thyroid hormones, lipids profile, blood parameters, and live weight Time: 13 weeks	↑ T3 in group 3: 1.65 (ng/mL) vs. 1.20 in control ↑ T4 in group 2 and 3: 24.85 (ng/mL), 27.35, respectively vs. 18.32 in control) ↑ final weight in group 3: 29.9 kg vs.14.6 kg in control	[75]
mustard (*Brassica juncea*) (high glucosinolate with content 24.6 mg/g DM of GLSs)	male lambs (14-day-old Avivastra) (Russian Merino Nali) (n = 24)	Groups: (1) control groundnut meal; (2) mustard meal Procedures: blood morphological parameters and measurement of the weight of internal organs Time: 26 weeks	mild thyroid enlargement and goiter in experimental group ↑ thyroid weight (g/100 kg): 15.01 vs. 10.69 in control	[76]
mustard meal (*Brassica juncea*)	male cross-bred (Jersey x Sahiwal) growing calves (230 days old and weight about 86.6 kg) (n = 24)	Groups: (1) control; (2) untreated mustard meal (25.5 mg/g dm GLSs); (3) HCl treated mustard meal (2.2 mg/g dm GLSs); (4) untreated mustard meal with iodine and copper supplementation (25.0 mg/g dm GLSs) Procedures: body weight and serum level of thyroid hormones were measured at 0 and 14 days of intervention Time: 2 weeks	no effect of iodine supplementation on T3 levels ↑ T4 (ng/mL) in group 4 (79.8) vs. control (63.32) plasma T3 concentration was found to be positively correlated with growth rate of calves	[73]
35% of mustard (*Brassica juncea*) cake concentration and ad libitum oat hay. Total GLSs content of the mustard cake was 5.9%	2-year-old goats (six intact and eight castrated bucks) (n = 14)	Groups: (1) bucks goats with 0 mg iodine in diet; (2) bucks goats with 0.1 mg iodine; (3) castrated goats with 0 mg iodine; (4) castrated goats with 0.1 mg iodine. All goats were fed with mustard cake. Procedures: measurement of thyroid hormones, lipids profile, blood parameters and live weight Time: 30 weeks	no effect of iodine supplementation on T3 levels ↑ T4 (ng/mL) between groups with and without iodine supplementation: 21.60 in group 1 vs. 28.41 in group 2 and 21.30 in group 3 vs. 30.70 in group 4	[72]
concentrate mixture containing mustard cake (12.79 μmol/g of GLSs)	crossbred goats (Alpine x Beetal) kids (4.5 months, 11.7 kg BW) (n = 12)	Groups: (1) control with untreated mustard cake (MC) diet (12.79 μmol/g of GLSs); (2) group with 1% CuSO_4_-treated MC diet (0.51 μmol/g of GLSs)—both groups were fed green maize and oats fodders ad libitum Procedures: measurement of thyroid hormones and histopathological analysis Time: 14 weeks	thyroid tissues of both groups didn’t show any morphological and histological changes no significant changes in thyroid hormones	[77]
crude extract of *Brassica juncea* L. seeds (mustard oil)	adult male rats (n = 32)	Groups: (1) control injected i.p. NaCl; (2) orally mustard oil; (3) injected i.p. 225 mg/kg CdCl_2_; (4) 112 mg/kg mustard oil daily after 1 h of CdCl_2_ Procedures: morphology and biochemical of blood tests (e.g., glucose, TSH, T_3_, T_4_, ALT, ASPT, lipid profile) Time: 3 weeks	↑TSH, T3, T4 in mustard oil group (0.73 ± 0.14 μg IU/mL, 1.91 ± 0.11 μg/dL, 2.06 ± 0.28 μg/dL, respectively) vs. control (0.50 ± 0.07, 1.50 ± 0.20, 1.40 ± 0.18, respectively protective effect of mustard oil against CdCl_2_	[71]
*Brassica carinata* meal pellet (28.7 μmol/g GLSs and allyl GLSs 26.86 μmol/g)	angus crossbred heifers (240 ± 39 kg initial body weight; BW) (n = 64)	Groups: (1) control (2) 0.3% of BW/d of *B. carinata* meal Procedures: Blood samples and body weight were collected weekly for 70 days. Plasma was analyzed for concentrations of triiodothyronine (T3), thyroxine (T4). Time: 10 weeks	no effect on thyroid hormone levels after 70 days	[74]
mustard seed oil (*Brassica juncea*)	male albino rats (n = 42)	Groups: (1) control; (2) hypothyroidism group; (3) hypothyroidism + mustard oil (1 mL/kg b.w.) group; (4) hypothyroidism + mustard oil (2 mL/kg b.w.); (5) hyperthyroidism group; (6) hyperthyroidism + mustard oil (1 mL/kg b.w.); (7) hyperthyroidism + mustard oil (2 mL/kg b.w.) Procedures: measurement of level of thyroid hormones, liver and kidneys parameters and assessment Time: 8 weeks	dose-dependent effect—reversal of the effect of hypo- or hyperthyroidism—higher dose is more effective ↑ fT3 (5.40 in 4 vs. 1.34 pg/dL in 2), ↑fT4 (4.46 ± in 4 vs. 1.74 ng/dL in 2), ↓ TSH (0.10 in 4 vs. 0.33 ng/dL in 2) in hypothyroid rats’ groups after mustard oil ↓ fT3 (7.66 in 7 vs. 13.50 ± pg/dL in 5), fT4 (7.23 in 7 vs. 11.37 ng/dL in 5), ↑ TSH (0.09 in 7 vs. 0.001 ng/dL in 5) in hyperthyroid rat groups after mustard oil administration	[70]

↑↓: Statistically significant increase or decrease in values.

#### 3.3.8. Radish

A noticeable influence on thyroid activity by radish root was observed in two studies on rats (Table 6). After administration of fresh or cooked roots, Chandra et al. noted symptoms of hypothyroidism in the animals, with an increase in TSH and a decrease in T3 and T4 values. The greatest change compared to the control group was observed in the TSH values for the group fed with fresh roots, where an increase of 286.4% was recorded. In both groups, there was also a decrease in thyroid peroxidase activity and an increase in thyroid weight. The fresh root had a stronger effect than the cooked one, probably due to the temperature decomposition of the active compounds [78]. However, Elnour et al. reached different conclusions, as the administration of ethanol extract from radish root led to a decrease in TSH values to zero after 2 weeks of use. Thyroid hormones did not show a uniform trend. The thyroxine level increased almost threefold, while the triiodothyronine level decreased [79]. Interestingly, no goiter was recorded in newborn lambs from ewes fed with fodder radish with a total GLSs content of 3 μmol/g, irrespective of iodine supplementation [80].

A sole study evaluated red radish sprouts, included in the diet of obese women as hypocholesterolemic and anti-obesity agents, but they also determined the TSH level in blood. Results revealed insignificant differences in the TSH level of all subjects before and after participating in this study [81].

**Table 6:** Goitrogenic potential of radish—animal and human studies.

Plant Material	Animals/Patients	Study Design	Results	References
		Animal Studies		
fresh *Raphanus sativus* Linn. (radish) (1.28 ± 0.4 mg/kg cyanogenic glucosides, 2.64 ± 0.2 mg/kg GLSs, 13.28 ± 0.9 mg/kg thiocyanate) and cooked radish (1.28 ± 0.4 mg/kg cyanogenic glucosides, 2.64 ± 0.2 mg/kg GLSs, 13.28 ± 0.9 mg/kg thiocyanate) (root)	Wistar rats (n = 60)	Groups: (1) control (laboratory diet); (2) fresh radish; (3) cooked radish; (4) laboratory diet + KI 12–14 μg/day; (5) fresh radish + KI 12–14 μg/day; (6) cooked radish + KI 12–14 μg/day. Procedures: histopathological evaluation, thyroid hormone levels, and urine examination. Time: 90 days	↓ T3, T4 levels and TPO activity in radish groups (2,3)—decline in the range 12.6–58.7% compared to control ↑ TSH level in radish groups (2,3)—growth in the range 210.2–286.4% compared to control. ↑ thyroid weight (g/100 g b.w.) in radish group vs. control greater impact for fresh radish than cooked radish similar results obtained in the KI groups ↑ hypertrophied follicles and small follicles in radish groups (2,3)	[78]
ryegrass, radish cv Graza (0.35 μmol/g glucoraphanin μmol/g, 1.9 μmol/g, glucobrassicin, 3.0 μmol/g of total GLSs) and rape cv Rangi (1.75 μmol/g progoitrin, 0.5 μmol/g 4-hydroxyglucobrassicin, 1 μmol/g glucobrassicin, 7.45 μmol/g of total glucosinolates)	newborn lambs (n = 204)	Groups: (1) ryegrass (control) without iodine; (2) ryegrass (control) with signal dose of 390 mg iodine; (3) radish without iodine; (4) radish with signal dose of 390 mg iodine; (5) rape without iodine; (6) rape with signal dose of 390 mg iodine Procedures: thyroid hormone (T3, T4) response to a thyroid releasing factor (TRF) challenge in ewes and incidence of goiter in newborn lambs was determined Time: 9 weeks	lack of an effect in newborn lambs fed with radish	[80]
*Raphanus sativus* (radish root)	adult male Wister albino rats (n = 28)	Groups: (1) control; (2) glibenclamide group (10 mg/kg b.w.); (3) ethanolic extract of radish (250 mg/kg b.w.) daily for two weeks Procedures: analysis of thyroid hormone levels, glucose, lipid and liver profiles Time: 2 weeks	↓ TSH in radish group after 2 weeks of treatment (1.30 ± 1.50 → 0.00 ± 0.00 ng/dL) ↑ T4 in radish group after 2 weeks of treatment (36.00 ± 6.00 → 110.00 ± 6.00 nmol/L) ↓ T3 in radish group after 2 weeks of treatment (0.70 ± 0.07 → 0.50 ± 0.07 nmol/L)	[79]
		Human Studies		
radish	technicians, medical students, and physicians (no data about health status) (n = 100)	Procedures: subjects with no breakfast, given a tracer dose of 100 microcuries of radioactive iodine in physiological saline; the uptake of radioactive iodine by the thyroid determined, followed by the instruction for the subjects to eat certain food such as cruciferous vegetables	lack of effect of raw radish on the iodine uptake	[7]
Red radish sprouts	females with body mass index (BMI) > 28 and aged between 25 and 40 years	Groups: (1) usual diet (n = 15); (2) low-calorie diet (n = 15); (3) low-calorie diet with radish sprouts (100 g per day) Procedures: anthropometric measurements, blood samples for analysis of TSH levels and other parameters Time: 8 weeks	before vs. after intervention: usual diet—TSH 1.89 ± 0.12 mIU/L, vs. 1.88 ± 0.09 mIU/L; low calorie diet—1.85 ± 0.19 mIU/L, vs. 1.85 ± 0.18 mIU/L; radish sprouts diet—1.91 ± 0.20 mIU/L, vs. 1.92 ± 0.10 mIU/L	[81]

↑↓: Statistically significant increase or decrease in values.

#### 3.3.9. Rapeseed

Rapeseed was the plant most frequently studied for its negative effects on the thyroid gland, probably due to its popularity as fodder. The detailed information about the possible influence of rapeseed products, mostly seeds, is presented in Table S3, due to the large number of studies about these seeds and the relatively low significant impact of these products available in the modern human diet on thyroid function. In this comprehensive description, the results of the study on rapeseeds’ impact on thyroid function in rats [82,83,84,85,86,87,88,89,90,91,92,93], dogs [94], cows [95,96,97], pigs and boars [98,99,100,101,102,103,104,105,106,107,108,109,110,111,112,113,114], lambs [80,115], and goats [116] were described. Only one study that focused on the iodized rapeseed oil’s impact on thyroid function in humans was found [27].

One of the first studies on this topic concerns the use of cyclical diets. Alternating use of a rapeseed diet, which leads to thyroid hypertrophy in rats, and a meat-based diet, which induces the involution stage of the thyroid gland, did not result in the permanent development of colloidal goiter or thyroid adenoma, in contrast to the constant use of a rapeseed diet alone, which resulted in the formation of adenomas [82].

Several studies clearly show that proper processing of rapeseed to remove substances that negatively affect the thyroid allows for their subsequent relatively safe use. Sulfur compounds commonly present in rapeseed and known to have a potential toxic effect include, among others, 5-vinyl-2-thiooxazolidone (VTO = goitrin), 3-butenyl isothiocyanate, gluconapin, and glucobrassicin [86,89,90,91]. The detoxification of rapeseed involves continuous 2 h extraction of rapeseed meal using water to remove isothiocyanates. This method can remove up to 97% of VTO [117]. Ballester et al. [83] noticed that the use of unprocessed seeds caused an increase in thyroid weight regardless of the sex of the rats and a decrease in body weight gain compared to the control. Such effects were not observed in the case of detoxified rapeseed presscake. However, in further studies, this form of rapeseed caused morphological changes in the thyroid, classified as intermediate between those in the control group (no changes) and those using unprocessed rapeseeds (enlarged thyroid gland with small follicles and a small amount of colloid). Water-extracted rapeseed meal containing small amounts of VTO (0.2%) had no effect on thyroid weight, unlike untreated rapeseed meal (VTO 5.5%). Untreated rapeseed also had a selective effect on thyroid hormones—it caused a decrease in T4 levels but had no effect on T3 and TSH [87]. In another study, the author [86] compared the harmfulness of a detoxified rapeseed meal with VTO, a substance naturally occurring in rapeseed seeds. The use of the pure compound significantly increased the mass of the thyroid gland, unlike rapeseed. Detoxified rapeseed had no effect on thyroid morphology or liver weight, unlike VTO.

Changes in thyroid activity may be influenced by the variety of rapeseed used, but also by the degree of food fragmentation (powdered or mashed), as well as the addition of some minerals to the diet. Vermorel et al. tested the ratio between the Jet Neuf and Tandem varieties of rapeseed. The greatest changes in thyroid hormone levels were observed when both varieties were used at an equal ratio of 40%:40%, with a significant, almost fivefold decrease in triiodothyronine and almost fourfold decrease in thyroxine when compared to the control group. In a further study, the same group of authors observed that the decrease in the levels of T3 and T4 hormones may be due to iodine and copper supplementation, as well as the ratio between the powder and mash forms of the rapeseed meal. The use of the mash form of rapeseed lowers the level of T3 and noticeably accelerates the growth of thyroid mass compared to the powder form of rapeseed. However, it seems that a higher dose of supplemented copper reduces excessive growth of thyroid mass [88]. In turn, zinc supplementation combined with a rapeseed meal may be a factor causing an increase in thyroid weight in rats compared to the control group. It should, however, be underlined that the effect was observed in the animals fed with high zinc dose (200 µg/g) or after a prolonged period of time in the experiment (16 weeks). In their further study, Shah et al. did not observe a significant effect of a high-fat rapeseed diet on thyroid hormone levels [84]. Nugon-Baudon et al. also used rapeseed meal with quantitatively determined content of active substances and observed a significant decrease in food intake, the level of T3 and T4 hormones, and an increase in thyroid weight in rats fed with rapeseed [90].

An interesting study described the influence of rapeseed meal (containing 36.7 µmol GLSs/g dry matter) on body and thyroid weight in germ-free rats, inoculated with different human bacterial flora prior to the dietary intervention. A noticeable decrease in body weight was observed in the groups inoculated with human fecal flora and *Escherichia coli* and fed with rapeseed seeds compared to the groups fed with soybean meal. Such effects were not observed in the group inoculated with the *Bacteroides vulgatus* isolate. It is suggested that the differential activity of the bacteria strains in the degradation of GLSs is responsible for this effect. At the same time, an increase in the weight of the thyroid gland and a decrease in T3 and T4 hormone levels were observed in all groups fed with rapeseed meal [91].

In animals other than rodents, the highest impact on thyroid hormones was found in pigs fed with 8% rapeseed and rapeseed meal. In boars, the decrease in thyroxine levels was up to 63.21% and 42.28%, respectively [98,100]. No significant changes in thyroid hormones were observed in lambs fed with canola meal, even at its highest (up to 75%) percentage share in the diet [115], nor in dogs or pigs [94,108]. Thyroid weight in pigs increased in most of the studies within the range of 45–80%, with the exception of two instances showing extreme changes of almost 300 and 400% in pigs fed with 15% rapeseed presscake meal [100].

Schone et al. evaluated the effect of a rapeseed meal rich in GLSs and copper and iodine supplementation on thyroid metabolism in pigs. GLSs-rich meal inhibited the synthesis of triiodothyronine but did not affect the level of thyroxine in the group supplemented with a dose of 500 µg/kg of iodine compared to the control. This was a much weaker effect than that in the groups taking methimazole and thiocyanate. This dose of iodine did not affect the amount of food consumed and the pigs’ body weight. Copper supplementation in a diet containing GLSs initially reduced their negative effect on triiodothyronine levels, but this effect disappeared after 17 weeks and there were still significant differences between groups [102].

Jahreis et al. examined the effect of rapeseed meal on thyroid function in pigs, in combination with iodine, copper, or zinc supplementation. The results showed a significant influence of iodine intake on mitigating the negative effects of thyrostatic substances. The weight of the thyroid gland was higher in pigs without iodine intake than in those supplemented with iodine. The thyroid hormones were affected by iodine presence in the diet. The thyroxine level was higher in the iodine group compared to the non-iodine intervention group. There were no significant differences between iodine (0.2/1 ppm), zinc (150 ppm), or copper (250 ppm) in terms of thyroid parameters [98].

#### 3.3.10. Rocket

Some interesting studies on rocket provide evidence of its protective effects on the thyroid glands in animals (Table 2). In mice with induced diabetes and hypothyroidism, 8 weeks of administration of rocket oil caused the thyroid follicles to return to an almost normal appearance, and the colloid content in the follicles was similar to that in the control group [52]. A protective effect of rocket was also observed in rats treated with abamectin, a compound with negative effects on the production of thyroid hormones, causing a decrease in thyroxine levels and an increase in TSH. The plant reversed the negative effects of this toxic compound. During treatment, not only was normalization of thyroxine and TSH levels observed, but also of free testosterone, progesterone, and estradiol [53]. Another study assessed the impact of a diet rich in rocket cake on hormonal parameters in calves. Different diet variants based on mustard cake and rocket cake contained a similar total amount of GLSs. However, the average values for thyroxine in the diet containing 25% arugula were significantly higher than those in the diet variants with 0 and 50% arugula. The authors suspect that the results obtained are due to the variability of the animals used in the study [51].

#### 3.3.11. Rutabaga

Two studies reported the impact of rutabaga, a less popular brassica vegetable, on the thyroid (Table 2). Rutabaga sprouts were included in the diet of rats with sulfadimethoxine-induced thyroid disorders. The sprouts deepened the declines in fT3 and fT3 in the sulfadimethoxine group. It should be mentioned that the consumption of rutabaga sprouts had no effect on thyroid hormones in the group without thyroid dysfunction [54].

Greer et al. observed in a human study that rutabaga exhibited significant antithyroid activity, as evidenced by the reduced uptake of radioactive iodine by the thyroid gland, when consumed raw, in quantities ranging from 280 to 363 g. This effect was observed whether the raw rutabaga was consumed as a puree or juice. Furthermore, when one subject ingested a purified extract of rutabaga equivalent to 2617 g of raw product, complete inhibition of iodine uptake was observed. The negative effect was not observed when the vegetables were cooked [7].

#### 3.3.12. Turnip

The goitrogenic potential of turnip was investigated in sole animal and human studies (Table 2). The increase in thyroid gland size was significant in lambs fed with turnip compared to the animals receiving additional iodine supplementation [55]. In a study by Greer et al. [7], it was observed that raw turnip inhibited the uptake of radioactive iodine by the thyroid in humans at a dose of 441 g, but no effect was observed when half that amount was consumed. Conversely, cooked turnip demonstrated a reduction in uptake in one test when 500 g was eaten, but no effect was observed in two other tests with 514 and 686 g.

### 3.4. Chemopreventive Potential of Brassica Vegetables and Their Active Compounds

Thyroid carcinomas represent a prevalent category among endocrine cancers, encompassing papillary thyroid carcinoma (PTC), follicular carcinoma, anaplastic thyroid carcinoma (ATC), and medullary carcinoma. While the majority of thyroid cancers are localized, and generally associated with favorable survival rates, there has been a noteworthy surge in the global incidence of thyroid cancer across all pathological types in recent decades [118]. This increase underscores the significance of investigating potential correlations between dietary factors and the risk of developing these cancers.

Some of the data obtained in survey studies (Table S2) suggest the relationship between the consumption of brassica vegetables and cancer; however, the results are inconclusive. Truong et al., in a case-control study found that high consumption of brassica vegetables (not specified) was associated with thyroid cancer among Melanesian women with low iodine intake [119]. Bandurska-Stankiewicz et al. studied the nutritional habits in the incidence of thyroid carcinoma in the Olsztyn province, Poland. The results suggest that regular intake of brassica vegetables was related to a 1.5-fold higher risk of thyroid carcinoma incidence when compared to the control group of healthy volunteers [120]. In contrast, a case-control study conducted in French Polynesia showed that high consumption of cabbage was not significantly associated with a decreased risk of thyroid cancer [121]. Moreover, another case-control study in Sicily by Fiore et al. indicated that consumption of brassica vegetables, such as cabbage, broccoli, and cauliflower was associated with a reduced risk of thyroid cancer. The authors also pointed out the appropriate need for the presence of iodized salt in the diet [122].

As mentioned previously, the compounds probably responsible for the chemopreventive properties of brassica vegetables are two sulfur compounds, namely SFN and I3C, intensively studied in terms of their impact on different cancers, including thyroid carcinoma. Although most of the studies are in vitro, their results are promising and also explain the mechanism of the positive impact on thyroid cancer cells.

Wang et al. investigated the therapeutic potential of SFN (2.5–10 µM) for different thyroid cancer cells (8305C, IHH4, FTC133, BCPAP, TPC1, K1) and elucidated the underlying mechanisms. The findings revealed that SFN significantly hindered thyroid cancer cell proliferation in a dose- and time-dependent manner, with IC_50_ ranging from 28.2 to 45.0 µM. In addition, it suppressed cell migration and invasion by targeting the epithelial-mesenchymal transition (EMT) process and key regulators, such as Slug, Twist, MMP-2, and MMP-9. SFN achieved these effects by inhibiting the phosphorylation of Akt, activating Erk and p38 signaling cascades and promoting mitochondrial-mediated apoptosis through a reactive oxygen species (ROS)-dependent pathway. The investigation of SFN’s role in cell cycle regulation indicated G2/M phase arrest, accompanied by altered expression of key cell cycle-related genes, such as Cdk1, Cdk2, CyclinB1, Cdc25C, Chk1, and p21. SFN-induced apoptosis was evident through increased levels of active caspase-3 and -9 and decreased levels of procaspase-3 and -9. SFN’s impact on cell migration and invasion was characterized by a dose-dependent reduction, accompanied by modulation of metastasis-related genes and EMT markers. SFN’s inhibition of the PI3K/Akt pathway and its downregulation of MMP-2 and -9 further contributed to its anti-metastatic effects. This study provided comprehensive insights into the cytotoxic potential of SFN in thyroid cancer, emphasizing its efficacy in inhibiting proliferation, inducing apoptosis, and suppressing metastatic features [123].

Sousa et al. focused on SFN and phenethyl isothiocyanate (PEITC) to assess their cytotoxic effects on anaplastic thyroid cancer human cell lines (T235, T238) in comparison with normal thyroid rat cells (PCCL3). SFN demonstrated varying IC_50_ values for T235 (26.1 mM) and T238 (1 mM) cells, indicating different sensitivity levels. PEITC exhibited similar IC_50_ values in both ATC (0.8–1.2 mM) and normal thyroid cells (0.6 mM). Additionally, the effects of cisplatin (10 mM), paclitaxel (1 mM), and doxorubicin (1 mM) were compared to the effects of PEITC (0.2, 0.5, and 1 mM). The combination of all aforementioned anticancer drugs with PEITC did not show a significant enhancement in the activity towards the tested cell lines [124].

An interesting study was performed on the potential role of SFN in photodynamic therapy (PDT), used in anaplastic thyroid cancer treatment. Chatterjee et al. combined photofrin-PDT with SFN to enhance PDT efficacy against human anaplastic thyroid cancer FRO cells. The investigation further explored different concentrations of photofrin (0–50 μg/mL) in the presence or absence of SFN (0–50 μg/mL) in dark conditions. SFN alone demonstrated no cytotoxicity against normal cells. However, the combination of SFN and photofrin-mediated PDT notably reduced FRO cells. This combination treatment synergistically induced cell cycle arrest through ROS generation and MMP depolarization. Significant modulation of Ras, MEK, ERK, and B-Raf proteins was observed due to the combination treatment. Apoptosis induction in anaplastic thyroid cancer cells was evident individually with PDT and SFN. However, their combined treatment resulted in a significantly higher apoptotic and anti-proliferative effect compared to individual doses. Microscopic observations confirmed the synergistic efficacy, revealing a higher percentage of cells undergoing apoptosis in the combination groups. The results demonstrated that the combination of SFN with PDT significantly and dose-dependently inhibited cell growth. The dose-response analysis indicated synergism, with the reduction in the SFN dose not affecting the overall activity. The study emphasizes the negative regulation of the Ras/MEK/ERK pathway as a key mechanism contributing to the enhanced efficacy of SFN in combination with PDT against anaplastic thyroid cancer [125].

Tadi et al. compared the anti-proliferative effects of I3C and DIM in various thyroid cancer cell lines, representing papillary and follicular carcinoma, along with primary human goiter cells. Four thyroid cancer cell lines (B-CPAP, 8505-C, CGTH-W-1, ML-1) and primary human goiter cells were treated with different concentrations of I3C and DIM (10, 25, 50, 100, and 500 µM). I3C demonstrated a dose-dependent reduction in cell viability, with IC_50_ values ranging from 300 µM to 500 µM, while DIM exhibited superior anti-proliferative effects, with IC_50_ ranging from 40 to 45 µM. ML-1 cells were a little less susceptible to DIM, with an IC_50_ of 50 µM. DIM induced a significant G1 arrest in the follicular thyroid cancer cell line ML-1, whereas no significant G1 arrest was observed in the papillary thyroid cancer cell line B-CPAP. DIM induced statistically significant apoptosis in follicular thyroid cancer cell lines (up to 4- to 5-fold higher than untreated samples), while papillary thyroid cancer cell lines exhibited comparable apoptosis levels with both DIM and I3C. The treatment of primary goiter cells with I3C did not significantly reduce the number of viable cells but altered cell morphology, suggesting cytotoxicity. DIM, at 50 µM, resulted in a decrease of over a 70% in viable cells within 72 h, indicating significant cytotoxic effects and decreasing the proliferation rate of the cells to almost 90%. This study underscores DIM’s superior effects compared to I3C in primary goiter cells, suggesting its potential as a chemopreventive agent in thyroid proliferative diseases [126].

In their further study, the authors used the same panel of thyroid cancer cell lines, expressing both isoforms of the estrogen receptor (ER), ER-α and ER-β. For subsequent analysis, the authors chose a concentration of 25 mM DIM. DIM exhibited cytotoxic effects by preventing the formation of cell clones, irrespective of estrogen treatment. This underscored the anti-estrogenic activity of DIM. Migration assays demonstrated that DIM significantly decreased the migration of thyroid cells by 37–57%, with the effect being cell line-dependent. Moreover, DIM prevented the enhancement of the migratory ability induced by estradiol, reinforcing its antiestrogenic characteristics. Scratch assays further revealed that DIM dose-dependently decreased cell migration by 50–60%. In adhesion assays, DIM significantly decreased cell adhesion by 58% (BCPAP), 42% (8505C), 70% (CGTHW-1), and 45% (ML-1). The combination of estradiol and DIM did not improve adhesion, suggesting DIM’s potential as an anti-estrogen/metastatic agent. The study also explored the invasive potential of the compound on thyroid cells. DIM significantly decreased invasion by 52% (BCPAP), 50% (8505C), 80% (CGTHW-1), and 48% (ML-1), highlighting its ability to counteract estrogen-enhanced invasive processes. Unfortunately, no normal thyroid cell line was used in the study, which could indicate the selectivity of the compound [127].

Two animal studies described the protective role of I3C on the carcinogenesis process in thyroid (Table S1). In the first one, allyl sulfide and I3C in a rat model of multi-organ carcinogenesis caused by diethylnitrosamine, N-methylnitrosourea, and N,N-dibutylnitrosamine were examined. The values for glutathione S-transferase placental positive (GST-P+) hepatic foci, which is a surrogate end-point marker for predicting liver carcinogenicity, were determined. In the group where three carcinogens were used, the mentioned value was 19.2 ± 9.2 no/cm^2^, while in the group with additionally administered allyl sulfide and I3C, it decreased to 9.5 ± 6.4, and 5.2 ± 2.3 no/cm^2^, respectively. A noticeable decrease was also noted in the incidence of thyroid adenomas in carcinogenic groups where allyl sulfide and carboxyethylgermanium sesquioxide were additionally administered. Importantly, in the histopathological examination, the administration of the compounds themselves proved to be safe [128]. In the second study, based on the same model of carcinogenesis, the administration of I3C resulted in an increase in the frequency of pathological changes in the thyroid glands observed during microscopic examination 52 weeks after the start of the experiment. After 24 weeks, no significant differences were observed between the groups. Interestingly, the compound, when administered alone, proved to be safe for rats [129].

Only a few studies have investigated the impact of the brassica vegetables on thyroid cancer cells in vitro, while no animal or human studies have been performed so far.

Broccoli sprouts revealed varied cytotoxic effects on FTC-133 and 8505 C thyroid cancer cells. The more metastatic FTC-133 cells displayed an increased response to the tested sprouts (IC_50_ 35.2 µg/mL), especially at lower concentrations, compared to the undifferentiated carcinoma 8505C cells (IC_50_ 114.1 µg/mL). Interestingly, no toxic effects for thyroid normal Nthy-ori 3-1 cells treated with broccoli sprout extracts were observed within the tested concentration range (0.025–0.3 mg/mL), even after prolonged exposure for up to 72 h. Viability of the cells remained over 60% after 24 h, decreasing to 56% and 54% after 48 and 72 h, respectively, at the highest concentration tested. The authors concluded that such high concentrations could be considered an acute toxic dose, unlikely to be achieved in the daily diet [39].

Similar studies were performed for kale sprouts, on the same cellular model. The sprouts were quantified towards the sulfur compounds, with sinigrin (37.6 mg/100 g dw), glucoiberin (25.4 mg/100 g dw), and I3C (64.1 mg/100 g dw) as predominant representatives. The kale sprout extracts showed no activity against follicular thyroid carcinoma FTC-133 and undifferentiated thyroid carcinoma 8505C but also showed no adverse effects on the viability of normal Nthy-ori 3-1 cells at concentrations as high as 0.5 mg/mL [130].

Regarding the fortified kale sprouts enriched with six newly synthesized organic selenium compounds, two of the compounds—E-NS-4 and EDAG-11—enhanced the cytotoxic activity of the sprouts against thyroid cancer cells. The sprouts enriched with E-NS-4 exhibited the highest cytotoxic effect, with comparable activity against both FTC-133 and 8505C (IC_50_ 80 and 80.14 µg/mL) cell lines. On the other hand, EDAG-11-enriched sprouts were cytotoxic to follicular carcinoma FTC-133 cells (IC_50_ 178 µg/mL), while undifferentiated thyroid carcinoma 8505C cells remained unaffected. The cytotoxic activity of selenium-enriched sprouts differed significantly in comparison to the non-fortified sprouts. Fortification with E-NS-4 also significantly increased the levels of I3C (64.1 vs. 135.9 mg/100 g dw), sinigrin (37.6 vs. 506.6 mg/100 g dw), glucoiberin (25.4 vs. 308.5 mg/100 g dw), DIM (7.0 vs. 11.5 mg/100 g dw), and SFN (3.7 vs. 1.1 mg/100 g dw), compared to non-fortified sprouts. Similarly, the fortification of kale sprouts with EDAG-11 showed an increase in concentrations of sinigrin, progoitrin, sulforaphane, and glucoiberin compared to normal sprouts. However, no correlations were noted between the content of GLS in the sprouts and their cytotoxic activity. The extracts showed no negative effects on the normal Nthy-ori 3–1 thyroid cells, confirming their safety [130].

## 4. Discussion and Limitations of the Studies

This systematic review focused on the available data regarding the influence of edible brassica plants on the thyroid in human and animal studies. Included in this review were mammals such as rodents, livestock (including cows, pigs, lambs, and goats), as well as boars, and even dogs. To delve deeper into the possible mechanisms of these vegetables and their active compounds, in vitro studies were implemented. Although the results of some animal studies indicate significant changes in the histopathological appearance of the thyroid gland or its weight after the consumption of brassica vegetables, their translation into real danger for humans is rather limited. Moreover, in a number of studies the protective role of brassica vegetables against some toxic compounds, or even their chemopreventive potential against the development of thyroid cancer, has been proven. In our opinion, these aspects should be thoroughly investigated in the future. Most of the studies demonstrating the goitrogenic effect of brassica plants were performed in the second half of the 20th century, with the animals being fed for an enormously long time, i.e., several weeks. Thus, the results of such prolonged exposure to brassica plants cannot accurately represent the real amount of vegetables and the frequency of their appearance in the human diet. Moreover, the majority of the studies were performed on rapeseed seeds, which contain high amounts of sulfur compounds, but are not consumed by humans in this form. In contrast, only a small number of studies were performed on brassica vegetables, which actually constitute a significant element of the human diet, e.g., broccoli, cabbage. We did not find any evidence about the influence of horseradish on the thyroid function.

Some issues should be identified as limitations or weaknesses of the studies cited in this review. The most important problem that should be raised is that, in most of the studies, the plants or extracts used were not quantified for the amount sulfur compounds, which makes their results questionable, or at least difficult to interpret. Moreover, the comparison of such results is almost impossible, as the difference in the content in GLSs among the species or varieties of the brassica plants may be really significant, as described in the literature. Another general weakness of the studies is that a vast number of the experiments lacks the proper identification of brassica vegetables used in the study—as some of the authors did not indicate the part of the vegetable (roots, bulbs, seeds, leaves, inflorescences) or its form (raw, cooked, powdered etc.). Additionally, some of the studies were performed on the mixture of the brassica plants and by-products, e.g., mustard/rapeseed cakes, or the forage with the addition of other plants with goitrogenic potential, e.g., millet. This may question the credibility and reliability of the results of such studies. The described animal models are often associated with simultaneous iodine deficiency in the diet or are performed in comparison to diets supplemented with iodine and other trace elements, such as copper or zinc. This blurs the final results obtained.

As far as the human studies are concerned, they were performed with a relatively small number of the participants, which does not allow valid final conclusions to be drawn. The participants were usually healthy volunteers, with no stated thyroid disorders, which prevents any speculation on the relationship between brassica vegetable intake and their influence on thyroid functioning. The two case studies indicating the goitrogenic effect of brassica vegetables were based on an irrational amount of the products in the daily diet, and thus cannot be translated into any recommendations regarding dietary restrictions for patients with hypothyroidism.

Finally, it should be underlined that there is no well-designed in vitro cellular thyroid model, that enables the assessment of positive and negative impacts of food or plant-derived compounds. This would help in the preliminary screening of the goitrogenic potential, but also the chemopreventive potential of the brassica vegetables used so far, the candidates for novel functional food from this group, and also the examples of superfoods, such as brassica hybrids, which are starting to be recognized for their attractive appearance and content of sulfur compounds, such as BrusselKale or broccolini. It would be also helpful to consider the safety potential of active compounds closely associated with brassica vegetables, such as indole 3 carbinol. Its classification as a novel food under European regulation highlights the importance of regulatory frameworks in ensuring food safety [131].

## 5. Conclusions

The presented studies cast doubt on previous assumptions that brassica plants have antithyroid effect in humans when consumed in reasonable and accessible amounts as part of a daily diet. The vast majority of the results indicate that brassica vegetables are safe for thyroid function, especially when the proper iodine supply is provided. However, it should be underlined that eating raw vegetables, especially in high amounts, may increase the risk of the negative impact on the thyroid, while the cooking process reduces this effect. To draw more far-reaching conclusions, more human research based on dietary interventions rather than questionnaires should be conducted.

## Figures and Tables

**Table 2:** Goitrogenic potential of Brussels sprouts, cauliflower, kohlrabi, rocket, rutabaga, and turnip—animal and human studies.

Plant Material	Animals/ Patients	Study Design	Results	References
		Brussels Sprouts		
fresh and cooked Brussels sprouts var. Roger and fresh Brussels sprouts var. Kundry	male Fischer rats (n = 344)	Groups in Experiment 1 (E1): (1) control (basal diet); (2) 15% uncooked Brussels sprouts diet; (3) 30% uncooked Brussels sprouts diet; (4) 15% cooked Brussels sprouts diet; (5) 30% cooked Brussels sprouts diet; (6) basal diet + KSCN 0.2% Groups in Experiment 2 (E2): (1) control (basal diet); (2) basal diet + KI 1.3 mg/kg DW; (3) 2.5% cooked Brussels diet; (4) 5% cooked Brussels diet; (5) 10% cooked Brussels diet; (6) 20% cooked Brussels diet; (7) 20% cooked Brussels diet + KI 1.3 mg/kg DW Procedures: histopathological evaluation and thyroid hormone levels and urine examination Time: 4 weeks	↓ T4 in all experimental groups in E1, the most in group 6 (19 ± 0.9 vs. 72.9 ± 2 mmol/L in control) ↑ TSH only in group 6 in E1 (6.1 ± 0.5 vs. 2.4 ± 0.3 mU/L in control). No change in E2 ↑ thyroid weight only in groups 3, 6 in E1 and group 5 in E2 ↑ total morphological activation in thyroids of group 6 in E1 and groups 5–7 in E2	[45]
Brussels sprouts	healthy subjects (n = 10)	Procedures: first phase—for 2 weeks normal diet, recording any consumption of brassica vegetables to establish baseline thyroid hormone concentrations; second phase—4 weeks of daily consumption 150 g of Brussels sprouts (220 mg of glucosinolates/100 g), boiled or steamed; third phase—2–3 weeks of exclusion of brassicas from diet. Blood samples were collected.	first phase—TSH 2.7 mIU/L, fT4 16.7 pmol/L, total T4 97.1 nmol/L, total T3 1.9 nmol/L second phase—TSH 2.6 mIU/L, fT4—16.8 pmol/L, total T4 95.7 nmol/L, total T3 1.8 nmol/L third phase—TSH 2.6 mIU/L, fT4—16.2 pmol/L, total T4 94.5 nmol/L, total T3 1.8 nmol/L	[46]
		Cauliflower		
cauliflower (*Brassica oleracea* var. *botrytis*) hydroalcoholic extract (fresh leaves) glucobrassicin, 58 ± 13 µmol/kg w.w. neoglucobrassicin)	male Wistar rats (n = 36)	Groups: (1) control; (2) sodium fluoride (NaF) (100 ppm); (3) cauliflower (100 mg/kg) +NaF; (4) cauliflower (200 mg/kg) +NaF; (5) cauliflower (400 mg/kg) + NaF; (6) cauliflower (400 mg/kg) Procedures: measurement of level of thyroid hormones Time: 30 days	↑ T4 (μg/mL) in plant control (group 6) vs. control (3.15 ± 0.11 vs. 2.52 ± 0.06) The most effective dose of extract was 200 mg/kg—↓ T3, T4 after fluoride poisoning	[47]
cauliflower	technicians, medical students, and physicians (no data about health status) (n = 100)	Procedures: subjects with no breakfast, given a tracer dose of 100 microcuries of radioactive iodine in physiological saline; the uptake of radioactive iodine by the thyroid determined, followed by the instruction for the subjects to eat certain foods, such as cruciferous vegetables	cooked cauliflower showed no effect	[7]
		Kohlrabi		
kohlrabi	rabbits of both sexes, aged 5–6 months, weight of average 2.2 kg (n = 14)	Groups: (1) kohlrabi 250 g daily; (2) control group alfalfa hay (each group n = 7). Procedures: thyroid condition found during operation and weight of thyroid gland Time: 5 weeks	twice normal size of thyroid gland-moderate hyperemia	[48]
kohlrabi consumed of 70–80 g/daily	guinea pigs (n = 12)	Groups: (1) control group with carrots; (2) kohlrabi; (3) kale (each group n = 4). Procedures: iodine concentration in thyroid gland after 24 h after feeding Time: 1 day	in kohlrabi group, lower accumulation of radioiodine in the thyroid gland (3343 ± 636 c.p.m./5549 ± 994 c.p.m. (1)); results may suggest goitrogenic effect of plants	[49]
kohlrabi sprouts (*Brassica oleracea* convar. *acephala* var. *gongylodes* L.)	Wistar rats (n = 72)	Groups: (1) control; (2) 7% kohlrabi sprouts diet; (3) iodine-deficient diet; (4) iodine-deficient diet with 7% kohlrabi sprouts; (5) standard diet with 0.025% SDM; (6) 7% kohlrabi sprouts diet with 0.025% SDM Procedures: tests of thyroid hormones and antioxidant levels, histopathology assay, hematological evaluation Time: 8 weeks	tendency for the protective effect of sprouts changes of TSH and T3 hormones in groups with thyroid dysfunction ↑ thioredoxin reductase (mU/mg) in kohlrabi sprouts (2) vs. control (5.89 ± 3.20 vs. 2.54 ± 0.13) ↓ TNF-α (pg/mL) in group 4 vs. group 3 (13.40 ± 6.24 vs. 71.67 ± 9.63) tendency to ↓ changes by the addition of sprouts in thyroid dysfunction groups of overall thyroid follicular epithelial, follicular luminal areas	[50]
		Rocket		
arugula (*Eruca sativa*) cake and mustard cake (sinigrin, gluconapin, glucobrassicanapin, glucoerucin)	crossbred male calves (n = 15)	Groups: (1) 100% mustard cake diet (18.19 µmol/g GLSs); (2) 75% mustard cake + 25% arugula cake (17.95 µmol/g GLSs); (3) 50% mustard cake + 50% arugula cake (17.59 µmol/g GLSs) Procedures: assessment of metabolism and hormone levels Time: 3 months	↑ average T4 level (mg/mL) in group 2 (42.01 ± 5.82) vs. other groups (26.50 ± 1.38 (group 1), 26.05 ± 8.08 (group 3)) no effect on T3 level	[51]
arugula (*Eruca sativa*) oil and crud of parsley (*Petroselinum crispum*) leaves	male albino mice (n = 20)	Groups: Groups 1–3 treated with a single dose of alloxan (AL) (100 mg/kg) and a daily dose of carbimazole (CAR) (0.6 mg/kg). (1) positive control; (2) arugula oil (0.02 mL/kg) group; (3) crud of parsley leaves group; (4) negative control Procedures: histopathological assessment Time: 8 weeks	the appearance of thyroid tissue after applying the oil and leaves was similar to the tissue in the untreated group	[52]
*Eruca sativa* (fresh whole plant)	male albino rats Sprague-Dawley (n = 18)	Groups: (1) control; (2) abamectin (1 mg/kg); (3) abamectin (1 mg/kg) + *E. sativa* (5 g/kg) Procedures: hormone measurement and histopathological analysis Time: 28 days	no effect on T3 level ↓ T4, ↑TSH (ng/mL) (2.10 ± 0.18, 2.66 ± 0.38, respectively) in group 2 vs. control (3.93 ± 0.52, 0.83 ± 0.64, respectively) protective effect of *E. sativa*—no differences in TSH and T4 levels between the control and group 3	[53]
		Rutabaga		
rutabaga (*Brassica napus* L. var. *napobrassica*) sprouts (212.4 ± 12.2 mg/100 g f.w. progoitrin, 154.2 ± 12.3 mg/100 g f.w. sinigrin, 334.2 ± 81.2 mg/100 g f.w. glucoerucin)	Male Fischer rats (n = 72)	Groups: (1) control; (2) 7% rutabaga sprouts diet; (3) iodine-deficient diet; (4) iodine-deficient diet with 7% rutabaga sprouts; (5) standard diet with 0.025% SDM; (6) 7% rutabaga sprouts diet with 0.025% SDM Procedures: tests of thyroid hormones and antioxidant levels Time: 8 weeks	no change in thyroid hormone levels between control and rutabaga sprouts group ↓ FRAP (μmol/L), GPX3 (U/mg) in rutabaga sprouts vs. control (446.3 ± 69.2 vs. 569.1 ± 79.7, 0.6 ± 0.1 vs. 0.4 ± 0.0, respectively) ↓ fT3 (pg/mL), fT4 (ng/mL) in rutabaga sprouts with SDM group vs. SDM group (0.4 ± 0.1, vs. 1.3 ± 0.1, 1.9 ± 0.3, vs. 3.8 ± 0.4, respectively)	[54]
rutabaga	technicians, medical students, and physicians (no data about health status) (n = 100)	Procedures: subjects with no breakfast, given a tracer dose of 100 microcuries of radioactive iodine in physiological saline; the uptake of radioactive iodine by the thyroid determined, followed by the instruction for the subjects to eat certain foods, such as cruciferous vegetables	Cooked rutabaga had no effect. Raw pieces of vegetable completely inhibited iodine uptake compared to only moderate effect of raw rutabaga puree.	[7]
		Turnip		
turnips (*B. rapa* L.)	crossbred Suffolk-Dorset lambs (50 wethers, 50 ewes, weighing 30 to 40 kg), (n = 100)	Groups in Experiment 1 (E1): (1) no supplementation; (2) iodine supplementation; (3) copper supplementation; (4) iodine plus copper. Groups in Experiment 2 (E2): (1) pasture as control; (2) kale-diet; (3) turnip-diet; (4) Chinese cabbage-based Procedures: postmortal extraction of thyroid gland; measurement of weight Time: 17 weeks	thyroid gland weight (+40.67% vs. control +10.2% (E1)) average body weight gain (+67 vs. control +89 g daily (E1)) confirmed enlargement was modest	[55]
turnip	technicians, medical students, and physicians (no data about health status) (n = 100)	Procedures: subjects with no breakfast, given a tracer dose of 100 microcuries of radioactive iodine in physiological saline; the uptake of radioactive iodine by the thyroid determined, followed by the instruction for the subjects to eat certain foods, such as cruciferous vegetables	Raw turnip had an inhibitory effect in a dose of 441 g Cooked turnip inhibited uptake in one test when 500 g was eaten but had no effect in two other tests with 514 and 686 g	[7]

↑↓: Statistically significant increase or decrease in values.

## Data Availability

Not applicable.

## Supplementary Materials

The following supporting information can be downloaded at: https://www.mdpi.com/article/10.3390/ijms25073988/s1.
