# Supplementary material for: Do Brassica Vegetables Affect Thyroid Function?—A Comprehensive Systematic Review
Source: Int J Mol Sci. 2024 Apr 3;25(7):3988. doi: 10.3390/ijms25073988 (PMC11012840; doi:10.3390/ijms25073988)
Supplement: Supplementary file 1 [file ijms-25-03988-s001.zip › ijms-2931146-supplementary.pdf]

Table S1. Goitrogenic potential of brassica sulfur compounds - animal and human studies.

Compound	Animal model/patients	Aim of the study	Study design	Results	References
ANIMAL STUDIES					
Cheirolin<br>goitrin<br>sinapin<br>thiocyanate<br>thiouracil<br>n-propyl isothiocyanate<br>methylothiouracyl (MeTU)<br>potassium thiocyanate<br>dicheirolin thiourea (DCTC)<br>NN'-diethylthiourea (DETU)	male rats Wistar<br>n=4 for each substance in acute tests;<br>n=5 in chronic tests for each substance	The influence of the compounds on thyroid.	Experiments: acute and chronic tests<br><br>Acute test: 6 first substances listed administered subcutaneous (1-5 mg/mL) assessing their antithyroid properties by acute ^{131}I uptake (IU)<br><br>Chronic test: MeTU, DCTC, DETU and cheirolin administered in drinking water for 21 days (0.05-0.1%) assessing antithyroid potential by IU, thyroid weight, and histopathology analysis	Acute test:<br>• ↓IU vs. control: cheirolin 9-43%; goitrin 9-11%; sinapin thiocyanate 69%; thiouracil 11%; n-propyl isothiocyanate 40%; methylothiouracyl 8%<br>Chronic test:<br>• ↓IU vs. control: DETU and MeTU 90 %<br>• significant histopathological changes – loss of colloid and marked hyperplasia.<br>• ↓IU by 16% vs. control for cheiroloin, raised epithelium and colloid reduction noted.<br>• No antithyroid activity of DCTC	[19]
Iodine (I)<br>1-methyl-2-mercaptoimidazole (TPA)<br>potassium thiocyanate (KSCN)	Yorkshire pigs (n=91)	The influence of goitrogens and iodine on the thyroid gland	Groups in Experiment 1 (E1): 1) casein-I 2) casein+I 3) soy-I 4) soy+I. TPA (0.075%) administered from day 1 to 21, I (0.2 ppm) from 22 to the end.<br>Groups in Experiment 2 (E2): 1) TPA-I 2) TPA+I 3) KSCN-I 4) KSCN+I. TPA (0.075%) or KSCN	• ↓ mass of thyroid (g/kg b.w.) in group with addition of I in E1 (0.27 (1) vs. 0.22 (2) and 0.35 (3) vs. 0.21 (4)).<br>• ↓ mass of thyroid in group with addition of I in E2 (0.34 (1) vs. 0.18 (2) and 0.42 (3) vs. 0.17 (4)).	[15]

			(0.5%) administered from day 1 to 28, I (0.2 ppm) from 29 to the end.<br>Groups in Experiment 3 (E3): 1) TPA-I 2) TPA+I (0.1 ppm) 3) TPA+I (0.2 ppm) 4) TPA+I (0.4 ppm) 5) TPA+TPA 6) control. TPA (0.075%) administered from day 1 to 31, I or TPA from 32 to the end.<br>Groups in Experiment 4 (E4): 1) KSCN-I 2) KSCN+I (0.1 ppm) 3) KSCN+I (0.2 ppm) 4) KSCN+I (0,4 ppm). KSCN (0.5%) administered from day 1 to 21, I from 22 to the end.<br>Administration: oral<br>Procedures: measurement of body weight, thyroid, and food intake.<br>Time: 42 days (E1), 47 days (E2), 60 days (E3), 40 days (E4)	• ↑protein bound iodine in group with addition of I in E1, E2.<br>• Comparable ↑ mass of thyroid/b.w. in groups with addition of iodine in E3.<br>• ↓ mass of thyroid (g/kg b.w.) in groups with addition of iodine in E4 (0.15 (1) vs. 0.09 (2-4)).<br>• ↓ iodine uptake (%) – dose-dependent effect – the lowest score for 0.4 ppm of I in E4 (35.7 (1) vs. 4.1 (4)).	
Ethylenebisisothiocyanate sulfide (EBIS)	male and female rats (n=180)	Effect of subacute EBIS poisoning	Groups: 0, 1, 10, 100, 1000 ppm of EBIS.<br>Administration: oral<br>Procedures: measurement of the weight of internal organs and body, analysis of thyroid hormones<br>Time: 30, 60, 90 days.	• No change in thyroid mass<br>• ↓T4 (μ%) (1.13 ± 0.68 vs. 4.88 ± 0.67) and ↓ I125 (%) (2.13 ± 1.16 vs. 3.73 ± 1.17) in 1000 ppm EBIS group after 7 days<br>• No significant difference between 0-100 ppm groups	[17]
Progoitrin sinigrin	male Wistar rats (n=100)	Antinutritional effect of selected GLSs	Groups: 1) progoitrin group 2) sinigrin group 3) glucobarbarin group 4) sinalbin group;	• ↑ thyroid weight in all groups compared to control	[13]

glucobarbarin sinalbin			concentration variants/each group: control, 0.2, 1, 5 mg/g DM and 1 mg/g with myrosinase 0.15 U/g DM<br>Administration: oral<br>Procedures: Measurement of the weight of internal organs and weight gain<br>Time: 9 days	• the largest thyroid mass in glucobarbarin group in dose 1 mg/g with myrosinase: 67.4 ± 3.05 μg/g vs. 21.0 ± 3.32 μg/g in control.	
phenyl isothiocyanate (PIT)	male Wistar rats (n=24)	Toxicity assessment of phenyl isothiocyanate	Groups: 0, 2, 5, 10 and 40 mg PIT/kg b.w/day for 5 times/week<br>Administration: gastric intubation<br>Procedures: Assessment of thyroid hormonal, liver enzymatic and hematological parameters, measurement of body weight and internal organs<br>Time: 4 weeks	• ↑no effect on thyroid weight in all doses.<br>• ↓ total T4 level in 40 mg/kg of PIT group (72 ± 25 nmol/l) vs. control (124±21 nmol/l)<br>• ↓ free T4 in 10 and 40 mg/kg of PIT groups (22±2, 16±4 nmol/l) vs. control (27±4 nmol/l).	[18]
gluconapin, epiprogoitrin glucoiberin	male Wistar rats (n=145)	Antinutritional effect of selected glucosinolates	Groups: 1) gluconapin group 2) epiprogoitrin group 3) glucoiberin group 4) glucoraphanin group 5) glucocheirolin group 6) glucotropaeolin group.	• no negative effect on thyroid in glucocheirolin, glucotropaeolin groups.	[16]

glucoraphanin<br>glucocheirolin<br>glucotropaeolin			Concentration variants/each group: control, 0.5, 2.5, 12.5 μmol/g DM and 2.5 μmol/g with myrosinase 0.15 U/g DW (except 12.5 μmol/g DM in glucoraphanin group)<br>Administration: oral<br>Procedures: Measurement of the weight of internal organs and weight gain<br>Time: 10 days	• ↓ thyroid weight in all doses in epiprogoitrin and glucoiberin group.<br>• ↓ thyroid weight (μg/g) in glucoraphanin group in dose 2.5 μmol/g (0.19 vs. 0.25 in control)<br>• ↓ thyroid weight (μg/g) in gluconapin in doses 2.5, 12.5 and 2.5+0.15 (0.16, 0.14, 0.13 respectively vs. 0.25 in control)	
allyl cyanide (ACN)<br>allyl isothiocyanate (AITC)<br>dimethyl disulphide (DMDS)	Scottish Blackface castrated male lambs (n=40)	Effects of oral intake of selected goitrogenic plant compounds on food intake and metabolism	Groups: ACN group (0 or 10 mmol/d),<br>AITC group (0 or 10 mmol/d) and DMDS group (0 or 25 mmol/d) daily<br>Administration: oral<br>Procedures:<br>Thyroid, liver and kidney function tests, analysis of cytochrome oxidase activity.<br>Time: 5 weeks	• T3 plasma level: ↑ in all treatment groups (but not significant)<br>• T4 plasma level (μmol/L): ↑ in all treatment groups - significant only in ACN group (83.9 vs. 72.2 in control)<br>• Free thyroxin index (%): ↑ACN group (22.9 vs. 20.6 in control) and DMDS group (22.1 vs. 21.4 in control, not significant)<br>• Blood GSH level (g/L RBC): ↓ ACN group (583 vs. 918 and AITC group (679 vs. 823 in control), ↑ DMDS group (826 vs. 676 in control, not significant)<br>• ↓ Food intake in all treatment groups.	[21]

benzyl isothiocyanate (BITC)	male Wistar rats (n=16)	Effect of benzyl isothiocyanate on thyroid and liver metabolism.	Groups: Untreated control, BITC group (10 mg/kg b.w. per day BITC as a single dose).<br>Administration: intragastric<br>Procedures: measurement of the weight of internal organs, thyroid hormones, and lipid metabolism<br>Time: 2 weeks	• ↓ T3, T4 levels (nmol/L): BITC group (0.94±0.09, 43.16±3.92, respectively) vs. control (1.25±0.03, 53.81±3.5, respectively)<br>• no difference in lipid profile	[20]
sinigrin (SIN)	mares (n=19)	The effect of sinigrin on thyroid hormonal parameters.	Groups: 1) control 2) low-SIN (20 mmol/day) 3) high-SIN (35 mmol/day).<br>Administration: oral<br>Procedures: Measurement of thyroid hormones and iodine in serum<br>Time: 12 weeks	• ↓ iodine level in serum in low-SIN and high-SIN groups vs. control after 12 weeks<br>• no difference in TSH level in all groups.<br>• T3 and T4 Delta Δ and fold change was ↑ in the control group than others.	[22]
allyl isothiocyanate (AICT)	rats (n=50)	Effect of allyl isothiocyanate	Groups: healthy control on standard diet and 4 groups with type 2 diabetes on high-fat diet	• no dose-dependent effect of AICT on thyroid hormone levels	

		on metabolism in diabetic rats	with concentrations of AICT: 0, 2.5, 5 and 25 mg/kg b.w. once a day<br>Administration: intragastric<br>Procedures: measurement of liver, thyroid, pancreas and kidney parameters<br>Time: 2 weeks	• ↓fT4 (pmol/L) in 5 mg/kg b.w. AICT group (11.29 ± 1.61 vs. diabetes group (13.93 ± 0.57)<br>• ↓fT3 (pmol/L) in 2.5, 25 mg/kg b.w. AICT groups (3.53 ± 0.37, 3.31 ± 0.89, respectively) vs. diabetes group (4.18 ± 0.35)<br>• thyroid gland mass – dose-dependent effect – the higher mass in 25 mg/kg AICT (3.65 ± 0.25 mg/100g b.w.)	[14]
			carcinogenesis model		
indole-3-carbinol (I3C)	male Sprague–Dawley rats (n=100)	Assessment of the impact of indole-3-carabinol in a rat multi-organ carcinogenesis model.	Groups: groups 1-2 sequentially treated with diethylnitrosamine (DEN) (100 mg/kg b.w., i.p., single dose), N-methylnitrosourea (MNU) (20 mg/kg b.w., i.p., four times, on days 5, 8, 11 and 14) and dihydroxy-di-N-propyl-nitrosamine (DHPN) (0.1% in the drinking water, during weeks 1 and 3). Animals of groups 1 and 3 administrated diet containing 0.25% I3C for 20 weeks after DMD treatment and given basic diet for 28 weeks.	• maximum body weight (g) in GE group (342 ± 87), minimum body weight in carcinogens C+I3C group (273 ± 79) vs. control (324 ± 82).<br>• no incidents of hyperplasia, adenoma, carcinoma of thyroid in AS, I3C, GE and control groups.<br>• ↓ incidence (%) of adenoma in C+AS (11), C+I3C (24), C+GE (11) groups vs. C group (50)	[128]

			Administration: intraperitoneal (DEN, MNU), oral (DBN, I3C)<br>Procedures: measurement of body and liver weight and histopathological analysis of internal organs<br>Time: 24 or 52 weeks		
sinigrin (SIN)	mares (n=19)	The effect of sinigrin on thyroid hormonal parameters.	Groups: 1) control 2) low-SIN (20 mmol/day) 3) high-SIN (35 mmol/day).<br>Administration: oral<br>Procedures: Measurement of thyroid hormones and iodine in serum<br>Time: 12 weeks	• no histological thyroid's effects in pure I3C group.<br>• ↑ frequency of histological findings in thyroid in carcinogens group after addition I3C (C+I3C) vs. pure carcinogens groups (C) after 52 weeks<br>• ↑adenoma/adenocarcinoma (%) in thyroid in C+I3C group (75%) vs. C group (29) after 52 weeks.<br>• body eight (g): C+I3C group 635.8 ± 85.8 vs. C group 622.1 ± 70.1 vs. I3C group 677.0 ± 74.0	[129]
HUMAN STUDIES					
Progoitrin	thyrotoxic (n=1) and euthyroid patients (n=7)<br><br>no data about gender and age	Evaluation of acute inhibition of radioiodine uptake	Procedure: 0.5 to 2.0 g of progoitrin administered orally. In five subjects, myrosinase given at the same time; in two the progoitrin incubated with myrosinase overnight at 37°C in a pH 5.6 phosphate buffer.	• ↓ grade of inhibition based on a scale of 0 = no effect and 5 = complete inhibition N=2: 0.5 g – 2g; N= 5: 2 g<br>• 24 h thyroidal accumulation of the iodine was 11 % after vs 71% before	[25]

Progoitrin	euthyroid subjects (n=7)	Evaluation of inhibition of the 24-hour radioiodine uptake	Procedure: progoitrin administered orally without myrosinase (0.1- 4.0 g); 24-hour iodine uptakes performed.	• mean value for the second 24-hour uptake was 111 + 1.9 % of the initial uptake	[25]
Progoitrin	healthy volunteers (n=23)	Second radioiodine uptake	Procedure: continuation of the study described earlier	• significant variability in response to a dose was observed.<br>• the second uptake ranged from 6.0 to 62.5% of the initial uptake for 2 g of progoitrin	[25]

Progoitrin	healthy volunteers (n=3)	Confirming the hydrolysis of progoitrin into goitrin in the large intestine	Procedure: Rectal administration of progoitrin (4 g dissolved in saline)	• in two volunteers, goitrin was detected in urine, and in one in the blood. More goitrin was excreted in the urine after rectal administration compared to oral<br>• one patient developed diarrhea after application and was removed from the study	[25]
Progoitrin	one patient with severe Graves' disease	Evaluation of the clinical efficacy of progoitrin as antithyroid agent	Procedure: progoitrin administered once daily (1 g) dissolved in orange juice; body weight (BW), serum protein-bound iodine (PBI), radioiodine uptake, basal metabolic rate (BMR) checked; the impact of a similar dose of propylthiouracil (300 mg) once daily examined for 9 days as reference model.	• progoitrin caused decline in BMR and PBI, increase in BW, started shortly after initiation of therapy.<br>• the 24-hour radioiodine uptake decreased from 77% to 39-44% during the progoitrin therapy.<br>• a similar uptake (39%) was observed after switched to propylthiouracil.	[25]

thiocyanates	healthy volunteer (n=37) (28 females, 9 males) aged between 16 to 54, smokers (n=32) and nonsmokers (n=5)	The antithyroid effect of thiocyanate	Procedure: thiocyanates administered with milk over a 12-week period with a daily intake of 8 mg; T4, T3, TSH and concentration of iodine in urine evaluated.	• thiocyanate in serum significantly increase in non-smokers (4.0 vs 7.0 mg/L).<br>• for 12 weeks no significant changes were noted in: (1) T4, T3, TSH and (2) concentration of iodine in urine in all volunteers	[23]
3,3'-diindolylmethane (DIM)	females with thyroid proliferation disorders (TPD) scheduled for a partial or total thyroidectomy (n=7)<br><br>phase I clinical trial study	Investigation of the antiestrogenic activity of DIM in TPD females	Procedure: DIM tablets (Bioresponse, Boulder, CO) (300 mg/day) for 14 days; all patients euthyroid. Thyroid tissues, urine, serum collected; pre- and post-DIM treatment serum and urine samples analyzed for DIM; DIM determined in thyroid tissue.	• increase in urine DIM was 383.5 ng/mg of creatinine.<br>• DIM increased significantly in serum (mean increase of 12.32 ng/mg of creatinine).<br>• DIM was detected in thyroid tissues with a mean concentration 40.67 ng of DIM/g of tissue (range 0.8–128.7 ng/g of tissue)	[26]
thiocyanates	participants aged 12 years or older included	The associations between	Procedure: multiple regression used to assess the relationships among TT4, FT4, TT3, FT3, TSH	• the negative associations between TT4 and thiocyanate, were evident	

	in the dataset for analysis. Exclusions comprised individuals with: (1) history of thyroid disease (2) current thyroid medication usage (3) pregnant women currently on estrogen therapy (n=1960) National Health and Examination Survey (NHANES) 2001–2002	urinary concentrations of iodide and perchlorate, nitrate, and thiocyanate, based on iodide uptake inhibition (IUI)	and thiocyanate; data extracted from the whole survey.	only in the subset with high urinary iodide levels, not in the subset with low urinary iodide levels	[24]

↑↓: statistically significant increase or decrease in values; DW – dry weight.

Table S2. Goitrogenic potential of brassica vegetables - questionnaire studies.

QUESTIONNAIRE STUDIES					
Plant	Patients	Aim of the study	Study design		References
Kale, turnip	primary school girls in Qom city of Iran	Determine the risk factors of goiter in primary school students	Groups: 1) girls without goiter (n=288); 2) randomly selected girls with goiter (n=284)<br>Procedures: 1050 girls from primary school examined for goiter. Questionnaire used to evaluate the risk factors of goiter.	Intake of goitrogenic food: controls - kale consumption 70.8%, turnip consumption 64.6%; girls with goiter - kale consumption 67.3%, turnip consumption 68%.	[29]
Cabbage, cauliflower, broccoli, turnip, radish	hypothyroid patients living in Jeddah	1) occurrence of iodine deficiency in hypothyroid patients 2) effect of diet rich in goitrogens on indices of iodine and thyroid status	Groups: 1) patients with confirmed hypothyroidism (n=53); 2) patients without diagnosis of thyroid diseases (n=53).<br>Procedures: blood and urine collected to study serum thyroid hormones, thyroid autoantibodies, thyroglobulin and urinary iodine concentration; dietary iodine and goitrogenic food intake assessed by questionnaire.	Results: controls - 1.55–3.18 μIU/mL, fT3 3.99–5.02 pmol/L, fT4 13.59–16.39 pmol/L; hypothyroid patients - TSH 1.22–5.44 μIU/mL, fT3 3.88–5.1 pmol/L, fT4 13.69–18.05 pmol/L. Intake of goitrogenic food: controls - cabbage 45%, cauliflower 38%, broccoli 17%, turnip and radish 9%; hypothyroid patients - cabbage 28%, cauliflower 21%, broccoli 2%, turnip 8 % and radish 11% (percentage of subjects consuming each vegetable per month).	[30]
Cabbage, brussels sprouts, cauliflower, broccoli, radish, turnip, swede, kale,	patients with thyroid carcinoma living in the Olsztyn province, Poland	Nutritional habits and addictions in the incidence of thyroid carcinoma	Groups: 1) patients with thyroid carcinoma (n=297); 2) healthy subjects (n=589).<br>Procedures: nutritional habits assessed by a questionnaire.	Intake of goitrogenic food: controls - infrequent intake ~79%, average ~20%, frequent ~1%; thyroid carcinoma patients - infrequent intake ~68%, average ~30%, frequent ~2%	[120]

horseradish					
Cabbage	patients diagnosed with differentiated thyroid cancer living in French Polynesia	Evaluation of dietary patterns and goitrogenic food consumption on thyroid cancer risk	Groups: 1) patients with thyroid carcinoma (n=229); 2) healthy subjects (n= 371).<br>Procedures: nutritional habits assessed by a questionnaire.	Intake of goitrogenic food: controls - 125 people ≤5 g/day, 125 people 6–15.5 g/day, 121 people ≥15.6 g/day; thyroid carcinoma patients - 74 people ≤5 g/day, 87 people 6–15.5 g/day, 68 people ≥15.6 g/day	[121]
Cabbage, cauliflower, turnip	school girls in the age of 11 – 16 years old living in Lahore (n=660)	Impact of dietary pattern on the thyroid status of adolescent girls' population in City District, Lahore.	Procedures: nutritional habits assessed by a questionnaire; urine and blood samples collected for urinalysis	Intake of goitrogenic food: daily intake of cabbage - 56 people, daily intake of cauliflower - 7 people, daily intake of turnip - 75 people	[31]
Cabbage	primary school children aged 6-12 years in Anchar district, Eastern Ethiopia (n=418)	Assessment of the prevalence of goiter and its associated factors	Procedures: children examined for the presence of goiter. Questionnaire used to assess feeding habits of the children.	Intake of goitrogenic food: 25.6% of the children consumed cabbage twice per week.	[32]
Cabbage, broccoli, cauliflower	patients diagnosed with thyroid cancer living	Evaluation of dietary habits and thyroid cancer risk	Groups: 1) patients diagnosed with thyroid cancer (n=106); 2) Healthy subjects (n=217).	Intake of goitrogenic food: controls - cabbage 20%, broccoli 19.8%, cauliflower 19.2%; thyroid cancer patients - cabbage 8.2%,	[122]

	in Sicily, Italy		Procedures: nutritional habits assessed by a questionnaire.	broccoli 12.7%, cauliflower 11.7% (% of people consuming each vegetable every day)	
Cabbage	patients diagnosed with hypothyroidism (n=500)	Evaluation of cabbage intake and occurrence of hypothyroidism	Procedures: not mentioned, probably nutritional habits assessed by a questionnaire	Intake of goitrogenic food: no data	[33]
Cabbage, cauliflower, radish and turnip	patients with suspected thyroid disorders living in Algeria (n=1098)	Relationship between the ingestion of glucosinolates rich food and the emergence of endemic goiter	Procedures: the subjects examined for the presence of a goiter. The determination of total glucosinolates for cabbage, cauliflower, radish and turnip bought in a local store. The average consumption of these vegetables assessed by a questionnaire.	Intake of goitrogenic food: the average consumption was 380.3 g per person a week, i.e. 54.32 g per person a day, and the weekly ingestion of glucosinolates per person was 369.4 μmoles, or 52.7 μmoles per day.	[34]
Cabbage, brussels sprouts, kale, cauliflower, broccoli. radish, kohlrabi, rutabaga, turnip, mustard	subjects living in Poland	Evaluation of the consumption frequency of goitrogenic foods in four different groups of people in Poland, on traditional, vegetarian, vegan, and pesco-vegetarian diets.	Groups: 1) traditional diet (n = 97); 2) vegan diet (n = 146); 3) vegetarian diet (n = 84); 4) pesco-vegetarian diet (n = 38).<br>Procedures: data collected using a questionnaire	Intake of goitrogenic food: Leafy brassica vegetables: 35.7 - 63.1% - never or occasionally; 2.1 - 6.2% - 1 bowl/dinner plate >1 x day. Broccoli and cauliflower: 42.8 - 65.8% - never or occasionally; 4.1 - 10.3% - 1 bowl/dinner plate 2-3 x week. Roots and tubers of brassica vegetables (radish, kohlrabi, turnip, rutabaga): 54.8 - 63.1% never or occasionally; 2.1 - 5.9% - 1 bowl/dinner plate 2-3 x week. Seeds of brassica vegetables: 17.8 - 26.3% never or occasionally; 3.1 - 10.3% 1 bowl/dinner plate >1 x day (% of respondents)	[35]
Cabbages, cauliflower, broccoli,	thyroid cancer patients	Investigation of the role of food rich in	Groups: 1) patients with thyroid cancer (n=293); 2) subjects without thyroid cancer (n=354).	Intake of goitrogenic food: European patients - 56.5 g/day; other ethnic groups - 51.7 g/day; Loyalty islands - 83.3 g/day; North	[119]

Brussels sprouts, turnips, and Swiss chard	living in New Caledonia for at least 5 years at the time of diagnosis	goitrogens and iodine in thyroid cancer	Procedures: nutritional habits assessed by a questionnaire.	Melanesian - 62.2 g/day; South Melanesian - 53.8 g/day	

Table S3. Goitrogenic potential of rapeseed - animal and human studies.

ANIMAL STUDIES					
Plant	Animal model	Aim of the study	Study design	Results	References
rapeseed meal	Wistar rats (n=67)	the influence of diet and iodine (potassium iodide) supplementation on pathological changes in the thyroid gland	Groups in Experiment 1 (E1): 1) control 2) rapeseeds diet + meat diet (5 cycles) 3) rapeseeds diet + meat diet (6 cycles) 4) rapeseed diet<br>Groups in Experiment 2 (E2): 1) rich iodine rapeseed diet 2) poor iodine rapeseed diet 3) poor iodine meat diet<br>Procedures: histopathological assessment<br>Time: 17-21 weeks	• no effect of cyclical diet changes on the thyroid condition in E1.<br>• ↑ thyroid weight (average 70 mg/100g) in group 4 in E1.<br>• ↑ number of thyroid adenomata in rapeseed diets – 16/26 rats in group 1, 16/23 rats in group 2, 1/18 rats in group 3 - in E2<br>• ↓ % number of adenomata in the iodine supplementation group (69.7% in poor iodine group vs. 61.5% in rich iodine group) in E2	[82]
20 or 40% rapeseeds flour (RSF) with very low residual glucosinolates content	beagle dogs, of both sex (n=18)	Evaluation of dietary rapeseed protein concentrate flours in dogs and their effect of thyroid function	Groups: 1) group received a semisynthetic complete diet with casein as the sole protein source 2) group received the same diet except that 20 % of the protein was supplied by RSF 3) group received the same diet except that 40% of	• only slight histological changes (fewer thyroid follicles per field in the two RSF-groups)<br>• no significant alterations of thyroxine level	[94]

			the protein was supplied by RSF (each group n=6)<br><br>Procedures: checked for radioiodine thyroid uptake, serum thyroxine concentration, thyroid weight, and histology Time: 13 weeks		
• detoxified rapeseed presscake (DRSM) meal (VTO 0.2 mg/g)<br>• crude rapeseed meal (RSM) (VTO 5.9 mg/kg, isothiocyanates 0.4 g/kg)	albino rats of both sexes (n=60)	assessment of the effect of detoxification of a rapeseed meal on the internal organs	Groups: 1) control (20% protein from casein) 2) 5% DRSM+15% casein 3) 10% DRSM+10% casein 4) 15% DRSM+5% casein 5) 20% DRSM 6) 20% RSM<br><br>Procedures: Assessment of body weight gain, weight of internal organs (e.g. liver, thyroid, kidneys) and histopathological evaluation.<br><br>Time: 4 weeks	• ↓ weight gain in RSM group (32.0±3.4 g) vs. control (95.2±6.3 g)<br>• ↑ male and female thyroid weight in RSM group (24.08±6.3, 19.00±8.3 g/100g b.w., respectively) vs. control (4.7±0.47, 4.82±1.39 g/100g b.w.)<br>• Enlarged thyroid with small follicles and a small amount of colloid in the RSM group. In all groups DRSM is an intermediate state between RSM and control.	[83]
• rapeseed meal (Tower and Turret) fed to dairy cows at 25% of the grain mixture	dairy cows-Holstein-Friesian at the declining stage of lactation (n=15) and Holstein-Friesian dairy	The effects of feeding rapeseed meals (RSM) on thyroid status, iodine and glucosinolate content of milk	Groups: 1) soybean meal for control 2) Tower RSM 3) Target RSM, each group (n=3)<br><br>Procedures: measurement of thyroid weight and	• Thyroid gland weight ↑(+49.01% vs control 0%)<br>• thyroid hormones↓(T4-25% vs control 3.3%, T3-18.56% vs control -2.32%)	[95]

• The content of VTO in Tower 12.2 µmol/g and in Turret 23.2 µmol/g	calves housed individually in calf rearing pens (n=9)	and other parameters were studied in dairy cows and young calves	thyroid hormones after the intervention<br>Time: 8 weeks		
rapeseed meal	male Wistar rats (n=32)	the influence of zinc (zinc sulfate) supplementation and the type of diet on the growth of internal organs	Groups in Experiment 1 (E1): 1) control 2) rapeseed diet + Zn (100 µg/g) 3) rapeseed diet + Zn (150 µg/g) 4) rapeseed diet + Zn (200 µg/g)<br>Groups in Experiment 2 (E2): 1) control 2) control diet + Zn (150 µg/g) 3) rapeseed diet + Zn (150 µg/g)<br>Procedures: measurement of the weight of internal organs<br>Time: 4 weeks (E1), 8 or 16 weeks (E2)	• the highest ↑ thyroid weight (g/100g b.w.) in group 4 vs. control (5.6±1 vs. 4.4±0.4) in E1.<br>• ↓ body weight in all rapeseed groups in E1.<br>• ↑ thyroid weight (g/100g b.w.) in group 3 vs. control (3.8 ± 0.4 vs. 2.7 ± 0.3) after 16 weeks in E2.	[84]
rapeseed (*Brassica napus,* cv. *Tower* and *Brassica campestris,* cv. *Span*)	male and female<br>Sprague-Dawley rats (n=120)	assessment of the impact of diet on thyroid hormones and mineral accumulation in internal organs	Groups: 1) control 2) laboratory chow diet 3) 10% Tower rapeseed meal diet 4) 20% Tower rapeseed diet 5) 10% Span rapeseed diet 6) 20% Span rapeseed diet	• no changes in weight of thyroid and I^{125} uptake between rapeseed groups and control<br>• no significant changes in T_3, T_4 levels	[85]

			Procedures: measurement of thyroid hormones, thyroid mass, and minerals in internal organs<br><br>Time: 16 weeks		
• water-extracted rapeseed meal (DRSM) (0.2% VTO)<br>• untreated rapeseed meal (RSM)	male rats (n=no data)	assessment of the effect of detoxification of a rapeseed meal on thyroid parameters	Groups: 1) control 2) DRSM diet 3) RSM diet<br><br>Procedures: analysis of TSH, T_3 and T_4 levels and histopathology of thyroid<br><br>Time: 10, 30, 60 days	• ↑ thyroid weight in RSM group4.<br>• ↓T_4 levels in RSM group.<br>• No changes in T_3 and TSH levels in all groups.	[87]
• 0.006 and 0.0012% VTO<br>• detoxified rapeseed presscake (DRSM) (0.005% VTO)	21-day-old albino rats of both sexes (n=40)	comparison of the effects of VTO and DRSM on internal organs and body weight.	Groups: 1) control (C), 2) 0.006% VTO (VTO6) 3) 0.0012% VTO (VTO12) 4) rapeseed meal with 0.005% VTO (R)<br><br>Procedures: Histopathological assessment and weight gain of internal organs<br><br>Time: 4 weeks	• ↑ body weight: 138.8 ± 6.4 g in VTO6 group vs. 116.4 ± 3.8 in R group after 4 weeks<br>• thyroid mass greater in groups VTO6 and VTO12 than in R and control groups<br>• the largest thyroid mass in males in VTO12 group: 8.7 ± 1.9 mg/100g vs. 3.5 ± 0.41 (C), 6 ± 1.81 (VTO6), 3.8 ± 0.73 (R)<br>• the largest thyroid mass in females in VTO12 group: 7.10 ± 2.07 mg/100g vs. 3.0 ± 0.92 (C), 5.2 ± 0.92 (VTO6), 3.3 ± 0.35 (R)<br>• changes in hyperactivity of thyroid in the histopathological assay in the VTO6 and VTO12 groups. In the VTO12 group, single lymphocytes were visible.	[86]

8% rape seed meal	pigs (weight at the beginning about 20 kg, at the end 90 kg) (n=42)	influence of phytogenic substances with thyreostatic effects in combination with iodine on the thyroid hormones in pigs	Groups: 1) soybean meal with 0.2 ppm iodine (I) 2) without I 3) 0.2 ppm I 4)1 ppm iodine 5) 1 ppm I +250 ppm Cu 6)1 ppm I +250 ppm Zn 7)1 ppm I +250 ppm Cu+150 ppm Zn (each group of n=6 and fed with 8% rape seed meal) Procedures: examined the weight of thyroid glands, thyroid hormones<br>Time: 14 weeks	• thyroid weight ↑526% (2), +36.18% (4), +1.3% (7) vs control<br>• thyroxine level -63.21% (2) -8.32% (3) +24.4% (4), -33.3% (7) vs 2.3% control<br>• T3 level +16.67% (2) -10% (4), +18.8% (7) vs 0% control<br>• phytogoitrogens lead to hypothyroid in pigs	[98]
rapeseed various Tandem (2.06 g/kg DM isothiocyanate, 4.88 g/kg DM VTO; 64 mM/kg DM glucosinolates)	male Sprague Dawley rats (n=100)	the effect of supplementation with iodine (potassium iodide), copper (copper sulphate) and iron (ferrous sulphate) on the level of thyroid hormones in the rapeseed diet	Groups: 1) control (powder 16 days/mash 19 days) 2) rapeseed diet (P35/M0) 3) rapeseed diet (P0/M35) 4) rapeseed diet + I^- (1 mg/kg) (P16/M19) 5) rapeseed diet + I^- (1 mg/kg) + Cu^{2+} (315 mg/kg) (P16/M19) 6) rapeseed diet + I^- (1 mg/kg) + Cu^{2+} (630 mg/kg) (P16/M19) 7) rapeseed diet + I^- (1 mg/kg) + Cu^{2+} (630 mg/kg) + methionine (1500 mg/kg) (P16/M19) 8) rapeseed diet + I^- (1 mg/kg) + Fe^{2+} (280 mg/kg) (P35/M0)	• ↓ T_3 (pg/mL) in groups 3, 4 (540±180, 410±110 respectively vs. 740±100 in control)<br>• ↑ T_3 (pg/mL) in group 6 (990±160 vs. 740±100 in control)<br>• ↓ T_4 (ng/mL) in groups 2, 3, 4 (22.9±4.5, 18.2±3.1, 16.5±2.5 respectively vs. 35.2±9.3 in control)<br>• ↑ thyroid weight (g/100g b.w.) in groups 2-5, 8, 9 (13.9±2.7, 18.7±4.3, 12.6±1.7, 9.6±2.1, 11.5±2.2, 10.2±1.3 vs. 4.7±0.4 in control)	[88]

			9) rapeseed diet + I^- (1mg/kg) + Fe^{2+} (560 mg/kg) (P35/M0) 10) rapeseed diet + I^- (1 mg/kg) + Fe^{2+} (560 mg/kg) (P0/M10) 11) control (P0/M10) 12) rapeseed diet + Fe^{2+} (560 mg/kg) (P0/M10)<br><br>Procedures: measurement of body weight, food intake, internal organs weight and thyroid hormone levels<br><br>Time: 35 days		
Rapeseed:<br>• Jet Neuf (151 mM/kg DM VTO, 28 mM/kg DM isothiocyanate, 210 mM/kg DM glucosinolates) and<br>• Tandem (23 mM/kg DM VTO; 8 mM/kg DM isothiocyanate,	male Sprague-Dawley rats (n=70)	the impact of diet on thyroid hormonal parameters	Groups: 1) control 2) 50% Tandem rapeseed diet 3) 100% Tandem rapeseed diet 4) 68% Tandem + 12% Jet Neuf rapeseed diet 5) 56% Tandem + 24% Jet Neuf rapeseed diet 6) 40% Tandem + 40% Jet Neuf rapeseed diet 7) 24% Tandem + 56 % Jet Neuf rapeseed diet<br><br>Procedures: measurement of body weight, food intake and thyroid hormone levels	• ↓ T_3, T_4 and body weight in all experimental groups<br>• Lowest value T_3 (pg/dl) in group 6 (10.6±0.6 vs. 48.2±6.6 in control)<br>• Lowest value T_4 (ng/dl) in group 6 (1.3±0.1 vs. 4.8±0.7 in control)	[89]

33 mM/kg DM GLSs)			Time: 22 days		
rapeseed meal (25 µmol/g DM progoitrin, 1.5 µmol/g DM gluconapoleiferin, 7.1 µmol/g DM gluconapin, 2.1 µmol/g DM glucobrassicanapin, 1.3 µmol/g DM 4-hydroxy-glucobrassicin, 0.3 µmol/g DM glucobrassicin)	male Fischer rats (n=44)	effect of food containing glucosinolates	Groups: soybean meal (SM), soybean meal + phenobarbital (0.75 g/l for first 19 days, 0.2 g/l for 12 days) (SM+PB), rapeseed meal (RM) and rapeseed meal + phenobarbital (0.75 g/l for first 19 days, 0.2 g/l for 12 days) (RM+PB).<br>Procedures: measurement of the weight of internal organs, thyroid hormones, and assessment of liver enzymatic activity<br>Time: 31 days	• ↓ food intake (g) in RM group (3333) vs. SM group (5068) within 27 days.<br>• ↑ thyroid mass (g/100g): RM group (11.5 ± 0.7) vs. SM group (3.8 ± 0.1)<br>• ↓ T_3, T_4 levels (nmol/l): RM group (1.33 ±0.04, 37±1.8, respectively) vs. SM group (1.97 ±0.09, 52.1±1.8, respectively)	[90]
rapeseed meal (winter type) contained 148 mmol GLSs and their degradation products (aglycones) per kg DM.	fattening pigs (Landrace x Large White) (20 kg initial body weight) (n=50)	effect of varying glucosinolate and iodine intake rapeseed meal diets on serum thyroid hormone level and total iodine in the thyroid in growing pigs	Groups: 1) soybean meal control group (n=6) 2) rapeseed meal + iodine (concentration of 0, 0.0625, 0.125, 0.25, 0.5, 1 mg) + treated with Cu (n=44 for other groups)<br>Procedures: all animals were treated with rapeseed meal groups versus control group with soybean meal.	• weight of the thyroid gland ↑(+512.5% in 0 mg iodine, +200% for 1 mg iodine and +12.5% for I+Cu vs 0% control).<br>• thyroxine↓(+42% for 0 mg iodine, -1.63% for 1 mg iodine, -40.38% for 0.5 mg Cu+1 mg I vs 37.2% in control)<br>• iodine supplementation led to improvement of parameters in thyroid	[102]

			The intervention includes evaluating the effect of different concentrations of iodine and with 0.5 mg Cu supplementation or without. Total iodine concentration was measured with thyroxine level and thyroid total weight.<br><br>Time: 15 weeks		
rapeseed meal (23.9 µmol/g DM progoitrin, 1.2 µmol/g DM gluconapoleiferin, 6.3 µmol/g DM gluconapin, 1.6 µmol/g DM glucobrassicanapin, 3,2 µmol/g DM 4-hydroxy-glucobrassicin, 0.5 µmol/g DM glucobrassicin 36.7 µmol/g DM glucosinolates)	male germ-free Fischer rats (n=36)	the influence of the vaccinated bacterial flora and the type of diet on the hormonal parameters of the thyroid gland and the weight of internal organs	Groups: 1) humane fecal flora (HFF) group – soy meal 2) (HFF) group – rapeseed meal 3) *Escherichia coli* (EC) group – soy meal 4) EC group – rapeseed meal 5) *Bacteroides vulgatus* (BV) group - soy meal 6) BV group – rapeseed meal<br><br>Procedures: measurement of the weight of internal organs and the level of thyroid hormones<br><br>Time: 7 weeks	• ↓ body weight (g) in HFF group fed rapeseed meal vs. soy meal (79 ±13 vs. 181±7) and in EC HFF group fed rapeseed meal vs. soy meal (53±9 vs. 187±11) after 7 weeks.<br>• ↑ thyroid weight in all groups fed rapeseed meal – the highest in BV group (242±20 vs. 36±2)<br>• ↓ T_3, T_4 in all groups fed rapeseed meal (without T_3 in 6 group)	[91]

rapeseed presscake meal (variety: Jet Neuf vs. Liradonna) containing relatively high amounts of GLSs and goitrin)	castrated boars (Large White breed 30-35 kg body weight) (n=42)	feeding of rapeseed presscake meal to pigs: effects on thyroid morphology and function and on thyroid hormone blood levels	Groups: 1) soybean as a control group 2) in the other 6 groups, soybean meal was substituted by 5, 10 and 15 % of either 0-(Jet Neuf) or 00-(Liradonna) rapeseed presscake meal (each group n=6)<br>Procedures: the blood sample was taken for thyroid hormones to evaluate changes<br>Time: 1 week	• in groups with 15% rapeseed meal ↓ thyroid hormones (T4 -42.82% vs -3.12% control, T3-16.22% vs -1.1% control)<br>• follicular and colloidal perimeters and epithelial thickness ↑ in the intervention group.	[101]
rapeseed presscake meal (15% in the ration) of 0-varieties (containing relatively high amounts of glucosinolates and goitrin)	castrated male pigs (Large White breed) with body weight about 25 kg (n=20)	effects of dietary rapeseed presscake meal on the thyroid activity in growing pigs	Groups: 1) rapeseed presscake meal (15% in the ration) of 0-varieties (containing relatively high amounts of glucosinolates and goitrin) 2) control diet (soybean meal instead of rapeseed presscake meal) without or with thyroxine added to feed.<br>Procedures: weight of thyroid was measure with thyroid hormones level of pigs	• weight of thyroid ↑ (+386% vs 0% control) in group 1<br>• thyroxine level ↓ (-28% vs -3.44% control) in group 1<br>• thyroxine supplementation has not affected thyroidal parameters	[113]

			Time: 7 weeks		
rapeseed presscake meal (RPM) of 0-varieties (containing relatively high amounts of GLSs and goitrin)	castrated boars (n=20)	growing animals fed rapeseed presscake meal and thyroid function	Groups: 1) 15% RPM of the 0-variety (0-RPM) 2) soybean meal as the main protein source 3) without or with supplemental L-thyroxine sodium salt (each group of n=5).<br>Procedures: body weight was measured along with blood sample for thyroid hormones.<br>Time: 7 weeks	• thyroid gland weight ↑in group 1 (+275% vs 0% control)<br>• daily gains were reduced in pigs fed rapeseed presscake	[100]
the rapeseed products originated from spring-sown, double-low cultivars of *Brassica napus* var. *oleifera* (Karat or Topas or Hanna).<br>The total GLSs content ranged from 17.6 to 31.0 μmol/g.	dairy cows of the Swedish Red and White breed (SRB) (n=85)	rapeseed products as feed for dairy cows. Effects of long-term feeding on thyroid function	Groups: 1) dietary groups with no rapeseed (NR) meal 2) up to 1.2 kg dry matter (DM) 00-rapeseed meal plus 0.2 kg DM full-fat 00-rapeseed (MR) 3) up to 2.5 kg DM 00-rapeseed meal plus 0.9 kg DM full-fat 00-rapeseed (HR) per day.<br>Procedures: thyroid function evaluated using the thyrotropin releasing hormone (TRH)-test and TSH performed on around	• TSH↑ in group 3 (57.06% vs 1.05% control)<br>• feeding dairy cows with rapeseed does not have negative effects on animal health or fertility	[96]

			90 and 300 days after calving.<br><br>Time: 43 weeks		
rapeseed (*Brassica napus* var. *oleifera*) GLSs at 18 mmol/kg DM	four groups of ten pigs (five female and five male castrated pigs with an initial live weight of 23 kg) (n=40)	to evaluate the effect of increasing dietary rapeseed press cake level on feed intake, live weight gain and the weight of thyroid	Groups: 1) 0 g/kg diet rapeseed press cake untreated 2) 50 g/kg diet rapeseed press cake untreated 3) 100 g/kg diet rapeseed press cake untreated 4) 150 g/kg diet rapeseed press cake untreated<br><br>Procedures: post intervention 111 days weight of thyroid was assessed<br><br>Time: 16 weeks	• thyroid gland weight ↑(+180% in group 4 group; +54.3% in group 2; +84.2% in group 3 vs 0% in control)	[103]
rapeseed meal with different content of GLSs (3.2-95 mmol/kg diet)	Crossbred pigs (German Landrace Large White Duroc) (20 female and 25 male castrates) (mean initial body weight of 22 kg) (n=45)	Rapeseed meal and/or iodine affect growth and thyroid. Investigations into GLSs tolerance in the pig.	Groups: 1) 5 (factor 1: dietary GLSs content)] 3 (factor 2: supplementary iodine 125/500 mcg/kg) factorial arrangement (each group n=9).<br><br>Procedures: thyroid gland weight was checked in respect of iodine	• thyroid gland weight ↑(+63.43% in non-iodine group; -1.27% in iodine group) vs 0% in control group<br>• iodine supplementation might reduce the effect of formation of goiter only in case of low amount of rapeseed meal	[102]

			supplementation Time: 8 weeks		
RSM (*Brassica campestris safiua* cv. *Kulru*) used was first solvent-extracted as normal and then subjected to heat-moisture-treatment. Very low GLSs content (2.9 pmol/g defatted meal)	healthy growing pigs (74 were of the Finnish Landrace (L) or Yorkshire (Y) breed, 80 were L X Y crosses and eight were offspring of L x Y sows and Hampshire boars). The average initial weight of the pigs was 25.3 kg (n=160).	to determine rapeseed meal (RSM) with a very low GLSs content as a substitute for soya bean meal (SBM) in pig feeding	Groups: 1) control group-soyabean meal 2) 33% replaced by rapeseed meal 3) 66% replaced by rapeseed meal 4) 100% replaced by rapeseed meal (each group n=40)<br>Procedures: the thyroid weight checked after intervention<br>Time: 12 weeks	• no significant change in thyroid weight due to a very low GLSs content	[104]
rapeseed meal (content 10 or 12%)	boars (Polish Landrace x 9 Polish Large White) of 42-45 kg body weight (n=48)	determine morphological changes in thyroid gland of boars consuming diets containing 10% rapeseed meal or 12% rape seeds over an extended period	Groups: 1) control group with soya-based 2) 10% rapeseed meal 3) 12% rapeseed meal (each n=16)<br>Procedures: weight of thyroid gland measurement<br>Time: 2-year	• thyroid gland weight ↑(+64.12% vs 23.12% in control)<br>• flattening of glandular epithelium and enlargement of colloid masses were observed	[114]

rapeseed meal (RSM) contained gluconapin, glucobrassicanapin, progoitrin, pronapoleiferin, indolylglucosinolates, 3-butenylisothiocyanate, 2-hydroxy-3-butenylnitrile	pigs (German Landrace x Large White x Duroc) (n=81)	assessment of pig tolerance to dietary glucosinolates and the effect of copper (copper sulphate) and iodine (potassium iodide) supplementation	Groups in Experiment 1 (E1): 1) control 2) cRSM (concentrate, high glucosinolate, 17.6 mmol/kg DM) + Cu 3) cRSM (135.8 mmol/kg DM) - Cu 4) nRSM (neutralized -low glucosinolate, 4.5 mmol/kg d.m) + Cu 5) nRSM (48 mmol/kg DM) - Cu Additional iodine supplementation in doses 62.5, 125 or 250 µg/kg.<br><br>Groups in Experiment 2 (E2): 1) control 2) methimazole 250 mg/kg 3) Thiocyanate (1000 mg/kg) 4) cRSM (9.5 mmol/kg glucosinolate) for 56 days. Additional iodine supplementation in doses 0, 125 or 500 µg/kg.<br><br>Procedures: measurement of thyroid hormone levels, body weight and food intake<br><br>Time: 56 days (E1), 119 days (E2)	• ↓ Body weight gain in cRSM-Cu group (487 g/day) vs. control (729 g/day) in E1.<br>• ↓serum T4 and ↑ thyroid mass after 17 weeks dependent on iodine dose and glucosinolate in diet – most effective in dose 62.5 µg/kg iodine and the highest of glucosinolate in E1.<br>• ↑ Body weight gain in cRSM group – dose-dependent effect – the highest score for 500µg/kg in E2.<br>• ↓T_4 in glucosinolate group for 125 and 500 µg/kg iodine vs. control in E2.<br>• ↓T3 in glucosinolate group without added iodine vs. control in E2.	[102]

rapeseed and rapeseed presscake (9.5 mmol GLSs per kg diet)	crossbred pigs (PiCtrain x German Landrace x Large White) (with an initial body weight of 31.5 kg) (n=98)	examine the negative effects of rapeseed and rapeseed press cake in pig diets	Groups: 1) 200 g toasted soybean (control group) 2) rapeseed content 50 g/kg 3) rapeseed content 100 g/kg 4) rapeseed content 150 g/kg with supplemented iodine 0.25 mg<br>Procedures: blood sample taken for serum thyroxine; thyroid weight measurement after 105 days of intervention<br>Time: 15 weeks	• thyroid gland weight ↑(+47.6% in group 3; +45.6% in group 4) vs 0% in control<br>• thyroxine level↓(-32.11% in group 3 0% in group 4) vs -2.12% in control	[103]
rapeseed press cake (3.2 mmol GLSs per kg diet)	male castrated pigs (from Pietrain or Landrace sires and Large White x Landrace cross dams) (n=60)	effects on rapeseed-press cake GLSs and iodine on the performance, the thyroid gland status of pigs	Groups: 1) 3 x 2 factorial arrangement (factor 1: dietary rapeseed press cake content (75 g/kg diet in groups 3 and 4 and 150 g/kg diet in groups 5 and 6), factor 2: dietary I content 125 ug iodine per kg diet whereas the groups 2, 4 and 6 received 250 ug supplementary iodine per kg diet. with 10 pigs allocated to each group	• thyroid weight ↑69.4% in group 4 vs 0% in control<br>• thyroxine level ↓(-42.86% in group 4 vs -11.1% in control)	[105]

			Procedures: weight of thyroid measured and thyroxine level.<br><br>Time: 12 weeks		
6, 8, 10% of rapeseed meal variety 00-RPM-varieties which contain only 30.13 μM of GLSs/g	healthy pigs (crossbreds of Landrace, Large White and Pietrain), pigs reached approximately 22 kg of body weight, at the mean age of 75 days; each group consisting of 30 animals (15 castrated males and 15 non-castrated females) (n=120)	investigation of thyroid hormones in pigs fed increasing amounts of 00-RPM	Groups: 1) control group fed with 6% sunflower seeds 2) fed with 6% of rapeseed meal 3) fed with 8 % of rapeseed meal 4) 10% of rapeseed meal (each group n=30)<br><br>Procedures: Blood samples taken at the 42nd day of experiment and of the finishing on 112th day. The weight of thyroid measured and thyroid hormones. Daily weight gain measured as well.<br><br>Time: 16 weeks	• weight of thyroid ↑ (+59.9% in group 4 vs 0% in control)<br>• thyroid hormones were not affected	[106]
10% of treated rape cake (reduced GLSs content not specified in numbers)	newborn calves (hybrids of 60% of Czech Pied cattle, 30% Ayrshire cattle, 10% Red Holstein cattle) (n=45)	evaluate the effect of starter diet containing 10% of treated rape cake (TRC) with a reduced glucosinolate content on calf	Groups: 1) control group with standard diet 2) comparative group with standard protein-rich diet 3) experimental group with 10% rape cake (every n=15)	• no significant changes in thyroid hormones<br>• weight gain of calves ↑ on 90 days of experiment (+24.4 kg in group 2, +27.2 kg in group 3 vs +20.7 kg in control group)	[97]

		growth, development, and health	Procedures: histological examination of the collected thyroid glands and thyroid hormones with iodine serum concentration<br><br>Time: 13 weeks		
Genetically modified rapeseed (synthesizing high amounts of mid-chain fatty acids).<br><br>Contents of GLSs 13.2 isogenic/20.4 transgenic (μmol/g)	crossbred hybrid pigs (n=20)	GMO rapeseed effects in growing-finishing pigs on iodine concentration and thyroid weight	Groups: 1) regular diet (n=10) 2) GMO rapeseed diet (n=10)<br><br>Procedures: measurement of thyroid gland weight and total iodine concentration after intervention<br><br>Time: 6 weeks	• iodine concentration↓ (-29.43% vs +1.09% in control)<br>• thyroid gland weight↑ (15.55% vs +2.31% in control)	[107]
rapeseed oil	male Wistar rats (n=54)	the effect of a fatty diet on thyroid function	Groups: 1) 5% sunflower oil 2) 10% sunflower oil 3) 20% sunflower oil 4) 5% rapeseed oil 5) 10% rapeseed oil 6) 20% rapeseed oil 7) 5% palm oil 8) 10% palm oil 9) 20% palm oil<br><br>Procedures: tests of the level of thyroid parameters and cholesterol<br><br>Time: 3 weeks	• the highest activity of thyroid peroxidase in 20% rapeseed oil group.<br>• the lowest level of hepatic type I deiodinase in 20% rapeseed oil group.<br>• the highest body mass gain in high-fat diet groups	[92]

rapeseed oil	male Wistar rats (n=54)	the effect of a fatty diet on thyroid hormones	Groups: 1) 5% sunflower oil 2) 10% sunflower oil 3) 20% sunflower oil 4) 5% rapeseed oil 5) 10% rapeseed oil 6) 20% rapeseed oil 7) 5% palm oil 8) 10% palm oil 9) 20% palm oil<br>Procedures: tests of the level of thyroid parameters and cholesterol<br>Time: 3 weeks	• ↑ T_4 – dose dependent effect in rapeseed oil group – the highest level in 20% group<br>• ↓ free T_4 – dose dependent effect in rapeseed oil group – the lowest level in 20% group<br>• ↑ Reverse-T_3 – only in 20% rapeseed oil group	[93]
fodder rape (concentration of total GLSs in rape 7.45 μmol/g)	newborn lambs (n=204)	the effect of differences in GLSs profile of fodder radish and a brassica on ewe thyroid function and incidence of goiter in lambs	Groups: a 3 x 2 factorial study utilizing three diets (Italian ryegrass, fodder radish, rape) and two rates of iodine (I) supplementation (with, without) was conducted. Procedures: thyroid hormone (T3, T4) response to a thyroid releasing factor (TRF) challenge in ewes and incidence of goiter in newborn lambs was determined<br>Time: 9 weeks	• A mild goiter, based on thyroid weight: lamb weight ratio (g/kg) of >0.4, was recorded only in newborn lambs from ewes fed rape and the incidence was higher (75% vs 11%) in those without I supplementation	[80]

rapeseed-mustard cake (GLSs content 64.2 μmol/g DM)	Male Barbari goat kids of average live weight 11.1 ±0.77 kg) and age (~6 months) (n=18)	This study examined the effect of (*Brassica juncea*) cake on the growth and thyroid status in goat kids.	Groups: 1) control, kids fed with concentrate containing groundnut cake (GNC) 2) rapeseed-mustard cake (RMC)-dry, kids fed concentrate containing (RMC) replaced for GNC 3) RMC-sani, kids fed water-soaked RMC concentrate (in fresh drinking water at 1:3 w/v ratio for 12 h at room temperature) as mixed ration (sani) with known amount of part of daily allowance of wheat straw (each group n=6) Procedures: weight changes and blood sampling at 45 days intervals for thyroid hormones<br>Time: 26 weeks	• serum T4↓ (-5.82% vs -0.32% in control)<br>• no change in thyroid weight	[116]
10% rapeseed meal (*Brassica napus*) (total GLSs content 14.5 μmol/g)	Four batches of crossbred Large White x Landrace sows (n=24)	effects of feeding 10% rapeseed meal on the performance of sows and their litters	Groups: 1) 0% rapeseed meal (n=12) 2) 10% rapeseed meal (n=12)<br>Procedures: weight of animals were carried out every cycle and plasma	• no significant changes in thyroid hormones and weight of animals	[108]

			level of thyroxine over 3 reproductive cycles<br>Time: 15 weeks		
conventional canola meal or high protein canola meal with 20 or 30%.<br>The concentration of total GLSs 12.60 mmol/g.	pigs (with an initial body weight of 9.15 ± 0.06 kg) (n=492)	Effects of growth performance of weanling pigs fed diets containing canola meal.	Groups: 1) control group soya based 2) high protein canola meal 30% 3) conventional canola meal 4) high protein canola meal 20% 5) control soya-based + carbohydrase 6) high protein canola meal 30% + carbohydrase 7) high protein canola meal 20% + carbohydrase 8) conventional canola meal + carbohydrase (each group n=61)<br>Procedures: weight of thyroid gland was measured<br>Time: 3 weeks	• weight of the thyroid gland ↑ (+67.53 in group 4 vs +8.21% in control group)<br>• partial impact of the growth performance in pigs fed with diet high-protein canola meal	[109]
the expeller extracted canola meal (EECM)<br>The concentration of GLSs in EECM - 9.27 μmol/g, with values in	pigs ([Yorkshire × Landrace] × Duroc; balanced for sex) with an average initial mean body	effect of EECM on growth performance, organ weights, and blood	Groups: The experimental diets were a corn–soybean meal–based - basal diet with 1) 0% 2) 10% 3) 20% or 4) 30% of EECM (each group n=12)	• thyroid hormone↓ (T4-25.71% vs -2.21% in control group)<br>• weight of thyroid gland ↑ (232% vs 0% in control group)	[110]

the diets ranging from 1.02 to 2.75 µmol/g	weight of 19.9 ±1.60 kg) (n=48)	parameters of growing pigs	Procedures: weight of thyroid gland was assessed, and thyroid hormones was pointed at finishing experiment<br><br>Time: 4 weeks		
cold pressed canola cake (CPCC) (derived from *Brassica napus* seed).<br><br>Total GLSs 14.9 µmol/g.	Pigs, initial b.w. of 7.8 ± 0.9 kg; Large White-Landrace female (line 42) × Duroc male (line 280) (n=160)	to determine effects of CPCC in diets for nursery pigs on thyroid parameters	Groups: four experimental diets included a corn-soybean meal (SBM)-based basal diet containing 1) 0% 2) 20% 3) 30%, or 4) 40% of CPCC (each group n=40) Procedures: weight of thyroid gland was performed respectively with blood sample for thyroid hormones on the 0 and 30 days of intervention<br><br>Time: 4 weeks	• thyroid hormones ↓ in group 4 vs control group (T3-29.32 vs -4.3%, T4-19.37% vs -1.32%)<br><br>• thyroid weight↑ (+128% in group 4 vs 0% in control group)	[111]
canola-derived GLSs via cold-pressed canola cake (CPCC).<br><br>Total GLSs content 11.1 µmol/g.	pigs (initial BW: 7.1 kg), which had been weaned at 21 d of age (n=240)	to determine effects of reducing high-amylose cornstarch (HA-starch) on growth performance, organ weights	Groups: 1) four diets were a basal diet with CPCC at 0 or 40%, and with HA-starch at 0 or 40% in a 2 × 2 factorial arrangement (each group n=60) Procedures: at the end of the study, one pig from each pen was euthanized to determine	• Thyroid gland weight↑ (+64.16%- in 40% CPCC group vs. +7.32% in control group with 0% HA-starch)<br><br>• thyroid hormones↓ (T3 -23.94%- in 40% CPCC group vs -2.52% in control group with 0% HA-starch)	[112]

		blood thyroid hormone levels nursery pigs fed cold-pressed canola cake	organ weights, blood parameters, hindgut pH, and GLSs degradation products Time: 4 weeks	• the negative effects of dietary CPCC on thyroid gland functions of nursery pigs were alleviated by dietary HA-starch.	
25%, 50%, 75% canola meal (CM)	growing Barki male lambs (4–5 months of age) (n=24)	the impact of replacing cottonseed meal with canola meal on thyroxine function	Groups: 1) control group with 0% CM 2) three experimental groups where CM replaced with 25%, 50% and 75% of cottonseed meal<br>Procedures: blood checked for serum triiodothyronine and thyroxine<br>Time: 14 weeks	• no significant changes in thyroid hormones<br>• no changes in growth performance	[115]
			HUMAN STUDIES		
Plant	Patients	Aim of the study	Study design	Results	References
Iodised rapeseed oil (Brassiodol)	patients with different stages of goitrous swelling (n=80)	Impact of iodized rapeseed oil in eradication of severe endemic goiter	Groups: subjects divided into 2 cohorts of 40 people, 4 groups with 10 patients/each, with different stages of goitrous swelling. Procedures: subjects received oral supplementation with a single dose of iodised oil (2 mL of Brassiodol).	Results:<br>• before administration of iodised oil - TSH 4.02 μU/mL, fT4 1.1 ng/dL, fT3 2.41 pg/mL;<br>• after 5 days - TSH 6.45 μU/mL, fT4 1.0 ng/dL, fT3 3.21 pg/mL;<br>• after 30 days -TSH 1.13 μU/mL, fT4 1.9 ng/dL, fT3 2.7 pg/mL;<br>• after 90 days - TSH 1.12 μU/mL, fT4 1.8 ng/dL, fT3 2.31 pg/mL;	[27]

			Time: Clinical follow-up lasts for a year and on days 5, 30, 90,180 and 360. Urinary iodine measured daily for the 5 days after intake of iodized oil (days 1–5) and on days 30, 90, 180, and 360. Measurement of TT4, TT3, free fT4, fT3 and TSH..	• after 180 days - TSH 1.23 μU/mL, fT4 1.8 ng/dL, fT3 2.34 pg/mL;<br>• after 360 days - TSH 2.43 μU/mL, fT4 1.4 ng/dL, fT3 2.28 pg/mL	

↑↓: statistically significant increase or decrease in values; VTO - 5-vinyl-2-thiooxazolidone, DW – dry weight, b.w. – body weight
